# The impact of quantile and rank normalization procedures on the testing power of gene differential expression analysis

**DOI:** 10.1186/1471-2105-14-124

**Published:** 2013-04-11

**Authors:** Xing Qiu, Hulin Wu, Rui Hu

**Affiliations:** 1 Department of Biostatistics and Computational Biology, University of Rochester, 601 Elmwood Avenue, Box 630, Rochester, New York 14642, USA

## Abstract

**Background:**

Quantile and rank normalizations are two widely used pre-processing techniques designed to remove technological noise presented in genomic data. Subsequent statistical analysis such as gene differential expression analysis is usually based on normalized expressions. In this study, we find that these normalization procedures can have a profound impact on differential expression analysis, especially in terms of testing power.

**Results:**

We conduct theoretical derivations to show that the testing power of differential expression analysis based on quantile or rank normalized gene expressions can never reach 100% with fixed sample size no matter how strong the gene differentiation effects are. We perform extensive simulation analyses and find the results corroborate theoretical predictions.

**Conclusions:**

Our finding may explain why genes with well documented strong differentiation are not always detected in microarray analysis. It provides new insights in microarray experimental design and will help practitioners in selecting proper normalization procedures.

## Background

Microarray technology has been widely adopted in many genomic related studies in the past decade. Despite its popularity, it is well known that various technical noises exist in microarray experiments [1,2] due to the limitation of technology. As a remedy, many normalization procedures have been proposed to remove these systematic noises, thus improving the detection of differentially expressed genes. Some efforts have been made to evaluate different normalization procedures [3-6]. Interested readers are referred to [7,8] for background and more detailed reviews of normalization procedures.

Quantile normalization is perhaps the most widely adopted method for analyzing microarray data generated by Affymetrix GeneChip platform. Motivated by quantile-quantile plot, it makes the empirical distribution of gene expressions pooled from each array to be the same [3]. It is the default option of BioConductor [9], which is a very popular open source software for analyzing microarray data implemented in R[10], the *de facto* standard statistical computing language in the statistical research community. This algorithm is also used for normalizing Affymetrix exon arrays [11,12], Illumina BeadChip arrays [13-15], Illumina transcriptome sequencing (mRNA-Seq) data [16], Illumina Infinium whole genome genotyping (WGG) arrays [17], and Solexa/Illumina deep sequencing technology [18], etc. In addition, several other popular normalization procedures are variants of quantile normalization, such as the enhanced quantile normalization [19] and subset quantile normalization [20] designed for microarrays, and the conditional quantile normalization [21] designed primarily for normalizing RNA-seq data.

Rank normalization is an alternative to quantile normalization. It replaces each observation by its fractional rank (the rank divided by the total number of genes) within array [22,23]. This normalization procedure achieves robustness to non-additive noise at the expense of losing some parametric information of expressions.

After normalization, a pertinent statistical test such as Student’s *t*-test [24] is applied to these *normalized *gene expression levels. The resulting *p*-values are adjusted by a multiple testing procedure (MTP) in order to control certain quantity of per-family Type I error, such as family-wise error rate (FWER) [25-28] and false discovery rate (FDR) [29]. Differentially expressed genes are identified based on a pre-specified threshold of adjusted *p*-values. More detailed introduction of statistical methods for detecting differentially expressed genes can be found in [30-33].

Without compromising the control of type I error, better testing power can be achieved by either increasing sample size or improving the strength of gene differentiation effect (fold changes between different phenotypes). Sometimes large expected differential effects based on biological considerations are invoked as a reason to justify a microarray study with very small sample sizes.

In this study, we find that one cannot “trade” differentiation effects with sample size. When the sample size is small, the statistical power for a gene differentiation analysis will not reach 100% even when the effect size approaches to infinity. This counter-intuitive phenomenon is due to the nature of the normalization procedures, which alters both sample mean difference and pooled sample standard deviation of the normalized expressions. As a result, they both grow at most linearly as functions of effect size and their effects cancel out. Our findings provide new insights into microarray experimental design which may help practitioners in selecting appropriate normalization procedures.

## Methods

### Notations and biological data

#### Notations

We assume that all expression levels are log-transformed. For convenience, the words “gene” and “gene expression” are used interchangeably to refer to these log-transformed random variables. These genes are indexed by *i *= 1,2,…,*m*, where *m *is the total number of genes.

Let *c *= *A*,*B *be two different phenotypic groups. For simplicity we assume that the number of arrays in both groups are the same and denoted by *n*. Without loss of generality, phenotypic group *A *is set to represent the phenotype of interest (usually the disease or the treatment group) and group *B *the normal phenotype. So up (down) regulation of a gene refers to its over (under) expression in group *A*. We denote by $y_{\text{ij}}^{c}$ the observed expression level of the *i*th gene recorded on the *j*th array sampled from the *c*th phenotypic group. The *normalized* counterpart of $y_{\text{ij}}^{c}$ is written as $y_{\text{ij}}^{*c}$.

The mean and standard deviation of $y_{\text{ij}}^{c}$ are denoted by $E\left(y_{\text{ij}}^{c}\right)=\mu_{i}^{c}$ and $\text{var}(y_{\text{ij}}^{c})=\sigma_{\text{ic}}^{2}$, respectively. Their normalized sample counterparts are denoted by ${\bar{y}}_{i\cdot }^{*c}=\frac{1}{n}\overset{n}{\underset{k=1}{\sum }}y_{\text{ik}}^{*c}$ and ${\left({\hat{\sigma }}_{i}^{*c}\right)}^{2}=\frac{1}{n-1}\overset{n}{\underset{j=1}{\sum }}{\left(y_{\text{ij}}^{*c}-{\bar{y}}_{i\cdot }^{*c}\right)}^{2}$, respectively.

In practice, the true level of gene differentiation is not a constant. It depends on the biological settings. The variance of gene expressions is nor constant either — it depends on the accuracy of measuring instruments and the homogeneity of biological subjects, just to name a few factors. In terms of statistical power, the decrease of gene expression variance is equivalent to the increase of mean difference. For simplicity, we consider gene expression variance to be fixed and define the effect size, our analysis tuning parameter, to be the expected mean difference of the *i*th gene expression between two phenotypes $e_{i}:=\mu_{i}^{A}-\mu_{i}^{B}$.

We divide genes into three sets: 

• *G*_0_, the set of non-differentially expressed genes (abbreviated as NDEGs). For all *i *∈ *G*_0_, $e_{i}:=\mu_{i}^{A}-\mu_{i}^{B}=0$.

•

$$G_{1}^{+}$$,

the set of up-regulated genes. For all $i\in G_{1}^{+}$, *e*_*i *_> 0.

• *G*1-, the set of down-regulated genes. For all *i *∈ *G *1-, *e*_*i *_< 0.

The set of differentially expressed genes (abbreviated as DEGs) is the union of both up-regulated and down-regulated genes, which is denoted by $G_{1}=G_{1}^{+}\cup G_{1}^{-}$. We write the size of these gene sets by *m*_0 _= |*G*_0_|, $m_{1}^{+}=|G_{1}^{+}|$, *m*1- = |*G*1-|, and *m*_1 _= |*G*_1_|. Apparently $m_{1}=m_{1}^{+}+m_{1}^{-}$ and *m*_0 _+ *m*_1 _= *m*.

#### Biological data

The biological dataset used in this study is the childhood leukemia dataset from the St. Jude Children’s Research Hospital database [34]. We select three groups of data: 88 patients (arrays) with hyperdiploid acute lymphoblastic leukemia (**HYPERDIP**), 79 patients (arrays) with a special translocation type of acute lymphoblastic leukemia (**TEL**) and 45 patients (arrays) with a T lineage leukemia (**TALL**). Each patient is represented by an array reporting the logarithm (base 2) of expression level on the set of 9005 genes.

### Analytic analysis of the impact of normalization procedures on differential expression analysis

In this section, we evaluate the impact of quantile and rank normalization on *t*-test. We are especially interested in studying the asymptotic property of the *t*-statistic as the effect size of differentiation approaches infinity while other parameters such as *n *and $\sigma_{i}^{2}$ are fixed. Empirical evidences in Section “Results and discussion” show that our findings are also valid for other statistical tests such as Wilcoxon rank-sum test and permutation based test.

To simplify theoretical derivation, we assume that the mean expression levels in the normal phenotype (group *B*) are zeros ($\mu_{i}^{B}=0$). This assumption implies that $\mu_{i}^{A}=\mu_{i}^{B}+e_{i}=e_{i}$. This simplification is reasonable because many hypothesis testing procedures such as *t*-test and Wilcoxon rank-sum test are invariant under shift transformation, so the mean difference between two groups, *e*_*i*_, is much more important than the normal level of gene expressions. For simplicity, we also assume that the effect size is a constant *e*^+ ^> 0 for all up-regulated and *e*^- ^< 0 for all down-regulated genes. In summary,

(1) $$E\left(y_{\text{ij}}^{c}\right)=\left\{\begin{array}{c} \;e^{+}\;c=A,\;i\in G_{1}^{+},\\ \;e^{-}\;c=A,\;i\in G_{1}^{-},\\ \;0\;c=A,\;i\in G_{0},\\ \;0\;c=B, \end{array}\right)$$

Therefore, the expected group differences of non-normalized gene expression data are

(2) $$E\left(y_{i\cdot }^{A}-y_{i\cdot }^{B}\right)=\left\{\begin{array}{ll} e^{+} & i\in G_{1}^{+},\\ e^{-} & i\in G_{1}^{-},\\ 0 & i\in G_{0}. \end{array}\right)$$

We must point out that all these assumptions are made only for the simplification of the theoretical derivations. Our findings essentially do not depend on these assumptions. This has been confirmed in our biological simulation study in Section “Results and discussion” (**SIMU-BIO**).

For the *i*th normalized gene expression, its *t*-statistic is defined as

(3) $$t_{i}^{*}=\sqrt{\frac{n}{2}}\cdot \frac{{\bar{y}}_{i\cdot }^{*A}-{\bar{y}}_{i\cdot }^{*B}}{{\hat{\sigma }}_{i}^{*}},$$

where ${\hat{\sigma }}_{i}^{*}=\sqrt{\frac{{\left({\hat{\sigma }}_{i}^{*A}\right)}^{2}+{\left({\hat{\sigma }}_{i}^{*B}\right)}^{2}}{2}}$ is called the pooled sample standard deviation.

The testing power of a two-sided *t*-test is determined by the absolute value of *t*-statistic. Based on Equation (3), it is clear that the testing power converges to 100% when *n* approaches infinity. For a fixed *n *(which also implies a fixed number of degrees of freedom), the testing power is determined by the absolute sample mean difference, $\left|{\bar{y}}_{i\cdot }^{*A}-{\bar{y}}_{i\cdot }^{*B}\right|$, and the pooled sample variance, ${\left({\hat{\sigma }}_{i}^{*}\right)}^{2}$. Below we study the asymptotic properties of these two quantities for quantile and rank normalized expressions separately.

#### Quantile normalization

With quantile normalization (**QUANT**), a reference array of empirical quantiles, denoted as **q **= (*q*_1_,*q*_2_,…,*q*_*m*_), is first computed by taking the average across all ordered arrays. Let $y_{(1),j}^{c}\leq y_{(2),j}^{c}\leq \cdots \leq y_{(m),j}^{c}$ denote the ordered gene expression observations in the *j*th array (*j *= 1,2,…,*n*) of the *c*th (*c *= *A*,*B*) group, the *r*th (*r *= 1,2,…,*m*) element of this reference array is

(4) $$q_{r}=\frac{1}{2n}\left(\overset{n}{\underset{k=1}{\sum }}y_{(r),k}^{A}+\overset{n}{\underset{l=1}{\sum }}y_{(r),l}^{B}\right).$$

The original expressions are replaced by the entries of the reference array with the same rank. Denote $r_{\text{ij}}^{c}$ as the rank of $y_{\text{ij}}^{c}$ in the array to which it belongs. The normalized gene expressions are

(5) $$y_{\text{ij}}^{*c}=q_{r_{\text{ij}}^{c}}=\frac{1}{2n}\left(\overset{n}{\underset{k=1}{\sum }}y_{(r_{\text{ij}}^{c}),k}^{A}+\overset{n}{\underset{l=1}{\sum }}y_{(r_{\text{ij}}^{c}),l}^{B}\right).$$

We refer the reader to [3] for more details.

In group *A*, over(under)-expressed genes tend to have high (low) ranks in each array. When the effect size is small, the ranks of DEGs in group *A *are mixed with those of NDEGs and the downstream testing power will be low. When the effect size is large, the DEGs in group *A *effectively take up all the top and bottom ranks, so the NDEGs in group *A *can only compete for ranks between *m*1- + 1 and $m-m_{1}^{+}$. We assume that the $m_{1}^{+}$ up-regulated genes almost always take the top $m_{1}^{+}$ ranks with equal chances and the *m*1- down-regulated genes almost always take the bottom *m*1- ranks with equal chances. We will show that the Student’s *t*-statistic of quantile normalized gene expressions follows a mixture distribution in which the doubly noncentral part converges to a distribution with finite all order moments instead of infinity when the true effect size becomes large.

We first investigate the asymptotic properties of sample mean difference ${\bar{y}}_{i\cdot }^{*A}-{\bar{y}}_{i\cdot }^{*B}$. Roughly speaking, quantile normalization ranks gene expressions first and then replace them by a reference quantile computed from all arrays. For an up-regulated DEG ($i\in G_{1}^{+}$), its rank can be among the top $m_{1}^{+}$ genes for all arrays in the normal group ($r_{\text{ij}}^{B}>m-m_{1}^{+}$, j=1,2,…,n) with probability $\left(\right)\frac{m_{1}^{+}}{m}{))}^{n}$. In this case, the expectation of sample mean difference is zero; otherwise it grows linearly as a function of *e*^+^. More specifically, by using conditional expectation, we obtain that for $i\in G_{1}^{+}$,

(6) $$\begin{array}{l} E({\bar{y}}_{i\cdot }^{*A}-{\bar{y}}_{i\cdot }^{*B}|r_{i1}^{B},\cdots \;,r_{\text{in}}^{B})\\ \propto \left\{\begin{array}{ll} O(1), & \text{with probability}{\left(\frac{m_{1}^{+}}{m}\right)}^{n},\\ O(e^{+},e^{-}), & \text{with probability}1-{\left(\frac{m_{1}^{+}}{m}\right)}^{n}. \end{array}\right) \end{array}$$

Similarly for down-regulated DEGs (*i *∈ *G*1-),

(7) $$\begin{array}{l} E({\bar{y}}_{i\cdot }^{*A}-{\bar{y}}_{i\cdot }^{*B}|r_{i1}^{B},\cdots \;,r_{\text{in}}^{B})\\ \propto \left\{\begin{array}{ll} O(1), & \text{with probability}{\left(\frac{m_{1}^{-}}{m}\right)}^{n},\\ O(e^{+},e^{-}), & \text{with probability}1-{\left(\frac{m_{1}^{-}}{m}\right)}^{n}. \end{array}\right) \end{array}$$

Detailed derivations can be found in Section 3 in the Additional file 1.

Similarly, ${\hat{\sigma }}_{i}^{*\cdot }$, the pooled sample standard deviation, can either grow linearly as a function of *e*^+ ^and *e*^- ^or (with positive probability) stay as a constant. Heuristically speaking, ${\hat{\sigma }}_{i}^{*\cdot }$ does not depend on *e*^+ ^or *e*^- ^if the ranks of expressions are all in the top group ($r_{\text{ij}}^{\cdot }>m-m_{1}^{+}$), middle group ($m_{1}^{-}<r_{\text{ij}}^{\cdot }\leq m-m_{1}^{+}$), or the bottom group ($r_{\text{ij}}^{\cdot }\leq m_{1}^{-}$) because all expression levels have the same effect sizes so they are canceled out. If the ranks are from different groups, some will have high expressions and some are low, the standard deviation will be “stretched out”. Since we assume up-regulated (down-regulated) genes in group *A *almost always take up the top (bottom) ranks, ${\left({\hat{\sigma }}_{i}^{*A}\right)}^{2}\propto \overset{n}{\underset{j=1}{\sum }}{\left(y_{\text{ij}}^{*A}-{\bar{y}}_{i\cdot }^{*A}\right)}^{2}$ does not depend on *e*^+ ^or *e*^-^. For group *B *we have

(8) $$\begin{array}{l} E\left({\left({\hat{\sigma }}_{i}^{*B}\right)}^{2}|r_{i1}^{B},\cdots \;,r_{\text{in}}^{B}\right)\\ \propto \left\{\begin{array}{l} O(1),\text{all}\;r_{i\cdot }^{B}\in \text{top}\;m_{1}^{+},\text{or middle}\;m_{0},\text{or bottom}\;m_{1}^{-}\\ \;\;\text{with probability}{\left(\frac{m_{0}}{m}\right)}^{n}+{\left(\frac{m_{1}^{+}}{m}\right)}^{n}+{\left(\frac{m_{1}^{-}}{m}\right)}^{n}\\ O({\left(e^{+}\right)}^{2},{\left(e^{-}\right)}^{2}),\text{otherwise with probability}\\ \;\;\;\;1-{\left(\frac{m_{0}}{m}\right)}^{n}-{\left(\frac{m_{1}^{+}}{m}\right)}^{n}-{\left(\frac{m_{1}^{-}}{m}\right)}^{n}. \end{array}\right) \end{array}$$

More detailed derivations can be found in Section 3 in the Additional file 1.

According to Equations (6), (7) and (8), the sample mean difference and pooled sample standard deviation both grow at most linearly as functions of *e*^+ ^(*e*^-^). As a result, the (absolute values of) *t*-statistics $t_{i}^{*}$ in (3) (given $r_{i1}^{B},\cdots \;,r_{\text{in}}^{B}$) for up-regulated DEGs ($i\in G_{1}^{+}$) approximately have the following mixture of central, noncentral and doubly noncentral forms:

(9) $$\begin{array}{l} t_{i}^{*}|r_{i1}^{B},\cdots \;,r_{\text{in}}^{B}\\ ∼\left\{\begin{array}{l} \frac{O(1)}{O(1)},\text{all}\;r_{i\cdot }^{B}\;\in \;\text{top}\;m_{1}^{+}\text{with probability}{\left(\frac{m_{1}^{+}}{m}\right)}^{n},\\ \frac{O(e^{+},e^{-})}{O(1)},\text{all}\;r_{i\cdot }^{B}\;\in \;\text{middle}\;m_{0}\;\text{or bottom}\;m_{1}^{-}\\ \;\;\text{with probability}{\left(\frac{m_{0}}{m}\right)}^{n}+{\left(\frac{m_{1}^{-}}{m}\right)}^{n},\\ \frac{O(e^{+},e^{-})}{O(e^{+},e^{-})},\text{otherwise with probability}\\ \;\;\;1-{\left(\frac{m_{0}}{m}\right)}^{n}-{\left(\frac{m_{1}^{+}}{m}\right)}^{n}-{\left(\frac{m_{1}^{-}}{m}\right)}^{n}. \end{array}\right) \end{array}$$

Similarly, the *t*-statistics $t_{i}^{*}$ for down-regulated DEGs (*i *∈ *G*1-) approximately have the following mixture forms:

(10) $$\begin{array}{ll} \; & t_{i}^{*}|r_{i1}^{B},\cdots \;,r_{\text{in}}^{B}\\ \; & ∼\left\{\begin{array}{l} \frac{O(1)}{O(1)},\text{all}\;r_{i\cdot }^{B}\;\in \;\text{bottom}\;m_{1}^{-}\text{with probability}{\left(\frac{m_{1}^{-}}{m}\right)}^{n},\\ \frac{O(e^{+},e^{-})}{O(1)},\text{all}\;r_{i\cdot }^{B}\;\in \;\text{middle}\;m_{0}\;\text{or top}\;m_{1}^{+}\\ \;\;\text{with probability}{\left(\frac{m_{0}}{m}\right)}^{n}+{\left(\frac{m_{1}^{+}}{m}\right)}^{n},\\ \frac{O(e^{+},e^{-})}{O(e^{+},e^{-})},\text{otherwise with probability}\\ \;\;\;1-{\left(\frac{m_{0}}{m}\right)}^{n}-{\left(\frac{m_{1}^{+}}{m}\right)}^{n}-{\left(\frac{m_{1}^{-}}{m}\right)}^{n}. \end{array}\right) \end{array}$$

To see this mixture under the normality assumption, we assume that all observed gene expressions $y_{\text{ij}}^{c}$ follow a normal distribution. Then, the normalized gene expressions $y_{\text{ij}}^{*c}$ approximately follow a normal distribution (See Section 2 in the Additional file 1). According to Equation (9), the *t*-statistics $t_{i}^{*}$ for up-regulated DEGs ($i\in G_{1}^{+}$) approximately follow a mixture of central, noncentral and doubly noncentral *t*-distributions with a density function

$$\begin{array}{ll} f_{t_{i}^{*}}\approx & {\left(\frac{m_{1}^{+}}{m}\right)}^{n}f_{t}+\left({\left(\frac{m_{0}}{m}\right)}^{n}+{\left(\frac{m_{1}^{-}}{m}\right)}^{n}\right)f_{T(\gamma )}\\ & +\left(1-{\left(\frac{m_{0}}{m}\right)}^{n}-{\left(\frac{m_{1}^{+}}{m}\right)}^{n}-{\left(\frac{m_{1}^{-}}{m}\right)}^{n}\right)f_{T(\gamma ,\lambda )}. \end{array}$$

Here *f*_*t*_, *f*_*T*(*γ*) _and *f*_*T*(*γ*,*λ*) _are the density functions of central, noncentral and doubly noncentral *t*-distributions, respectively, with *ν *= 2 *n *- 2 degrees of freedom. *γ *∝ *O*(*e*^+^,*e*^-^) is the numerator noncentrality parameter and *λ *∝ *O*((*e*^+^)^2^,(*e*^-^)^2^) is the denominator noncentrality parameter (from noncentral *χ*^2^) [35]. Similarly, according to Equation (10), the *t*-statistics $t_{i}^{*}$ for down-regulated DEGs (*i *∈ *G*1-) approximately follow a distribution with a density function

$$\begin{array}{ll} f_{t_{i}^{*}}\approx & {\left(\frac{m_{1}^{-}}{m}\right)}^{n}f_{t}+\left({\left(\frac{m_{0}}{m}\right)}^{n}+{\left(\frac{m_{1}^{+}}{m}\right)}^{n}\right)f_{T(\gamma )}\\ & +\left(1-{\left(\frac{m_{0}}{m}\right)}^{n}-{\left(\frac{m_{1}^{+}}{m}\right)}^{n}-{\left(\frac{m_{1}^{-}}{m}\right)}^{n}\right)f_{T(\gamma ,\lambda )}. \end{array}$$

**Figure 1:**
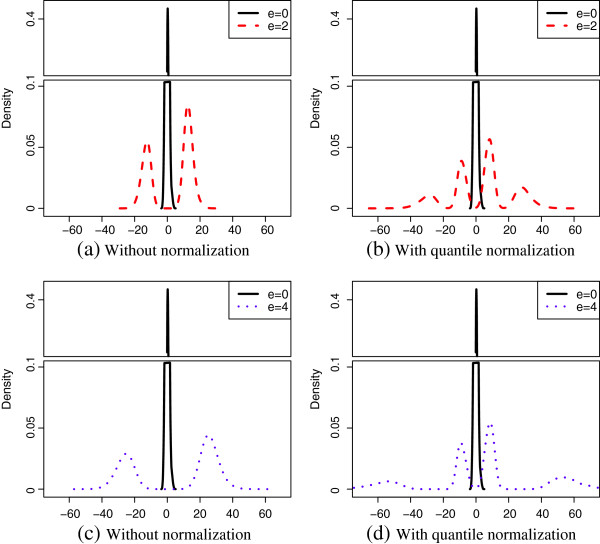
**Empirical density estimates of the *t*-statistics before and after quantile normalization. **Empirical density estimates of the *t*-statistics before and after quantile normalization. Gene expression are simulated by using normal random numbers with standard deviation 0.35 and gene-gene correlation 0.9. Total number of genes is *m *= 1000. Total numbers of truly differentially expressed genes are $m_{1}^{+}=60$ for up-regulated genes and *m*1- = 40 for down-regulated genes. The sample size is *n *= 10 and the true effect size is *e*^+ ^= -*e*^- ^= *e *= {0,2,4}. Estimates are based on 200 repetitions.

**Figure 2:**
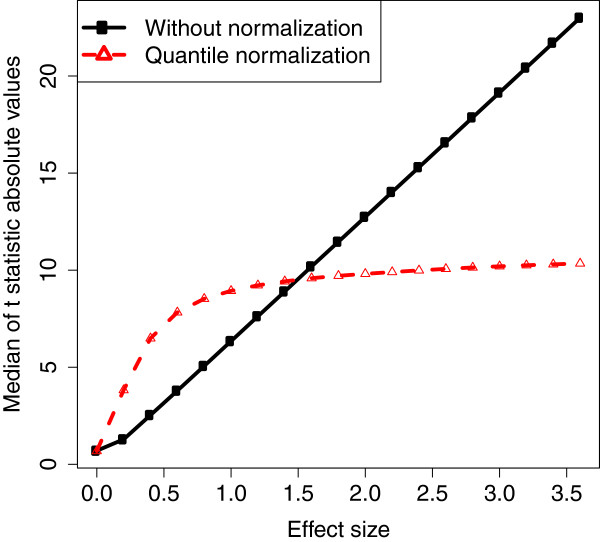
**Medians of the *t*-statistic absolute values. **Medians of the absolute values of the *t*-statistics for data with and without quantile normalization. Gene expression are simulated by using normal random numbers with standard deviation 0.35 and gene-gene correlation 0.9. Total number of genes is *m *= 1000. Total numbers of truly differentially expressed genes are $m_{1}^{+}=60$ for up-regulated genes and *m*1- = 40 for down-regulated genes. The sample size is *n *= 10 and the true effect size is *e*^+ ^= -*e*^- ^= *e *= {0,0.2,0.4, …,3.6}. Estimates are based on 200 repetitions.

In microarray analysis it is reasonable to assume *m*_1 _≪ *m*, i.e., the proportion of DEGs is small (*m*1- ≪ *m *and $m_{1}^{+}\ll m$). So the central *t*-distribution part in the mixture is negligible. Empirical density functions of $t_{i}^{*}$ for quantile normalized DEG expressions with different effect sizes are shown in Figures 1 (b) and (d). For effect sizes 2 and 4, the two peaks in the center represent the doubly noncentral *t*-distribution part *T*(*γ*,*λ*) and the two peaks to the far left and right sides represent the noncentral *t*-distribution part *T*(*γ*). The doubly noncentral *t*-distribution converges to a distribution with finite all order moments when *e*^+ ^(*e*^-^) approaches infinity. Figure 1 shows the convergence of $t_{i}^{*}$. Furthermore, we let *e*^+ ^and -*e*^- ^vary from 0 to 3.6 and the medians of the *t *statistic absolute values for DEGs with and without quantile normalization are plotted in Figure 2. Clearly, the median for data without normalization grows linearly while the median for data with quantile normalization is upper-bounded by a fixed constant when effect size becomes large. Therefore, the testing power associated with a two-sided *t*-test cannot reach 100%. The derivation of this convergence can be found in Section 4 in the Additional file 1. This result suggests that even if certain genes are known to have dramatically different expression levels for different phenotypes, a typical differential expression analysis based on quantile normalized expressions may not be able to detect them. In this case, combining the results obtained from differential expression analysis without normalization may provide new insight to the underlying biology.

Empirical evidences in Section “Results and discussion” also show that the statistical power converges to a fixed number strictly less than 1.0; and this convergence is independent of the hypothesis testing methods and MTPs being applied. Heuristically speaking, **QUANT **“borrows” information from both NDEGs and DEGs to reduce data variation, and as a result the normalized expressions are complex *mixture *of both NDEGs and DEGs with possibly very high true group differences. Consequently, the variances of normalized DEGs are asymptotically dominated by the differences between the NDEGs and DEGs and become increasing functions of effect sizes. Asymptotically, the increased variances cancel out the contributions of the increased effect sizes to the testing power.

#### Rank normalization

With rank normalization (**RANK**), we replace each entry in one array by its position (rank) in the ordered array counted from the smallest value divided by the total number of genes. Denote $r_{\text{ij}}^{c}$ as the rank of $y_{\text{ij}}^{c}$ in the array to which it belongs, the normalized gene expressions are

(11) $$y_{\text{ij}}^{*c}=\frac{r_{\text{ij}}^{c}}{m}.$$

This method was proposed by [22] and discussed further in [23].

Compared with **QUANT**, **RANK **goes even further in the nonparametric direction. It removes the noise by only preserving the ordering of observations. We know *m *is usually very large in a typical microarray study. If the effect size is large such that the over-expressed genes always take up the top $m_{1}^{+}$ ranks and the under-expressed genes always take up the bottom *m*1- ranks in group *A*, $y_{\text{ij}}^{*c}$ approximately has the following uniform distribution:

(12) $$y_{\text{ij}}^{*c}∼\left\{\begin{array}{ll} U(1-\frac{m_{1}^{+}}{m},1), & c=A,\;i\in G_{1}^{+},\\ U(0,\frac{m_{1}^{-}}{m}), & c=A,\;i\in G_{1}^{-},\\ U(\frac{m_{1}^{-}}{m},1-\frac{m_{1}^{+}}{m}), & c=A,\;i\in G_{0},\\ U(0,1), & c=\mathrm{B.} \end{array}\right)$$

Here for simplicity, again we assume that the genes take the specified ranks with equal chances within each group. Therefore, the normalized gene expressions no longer depend on the effect size. The expected group differences for rank normalized genes are

(13) $$E\left(y_{i\cdot }^{*A}-y_{i\cdot }^{*B}\right)\approx \left\{\begin{array}{cc} \frac{1}{2}-\frac{m_{1}^{+}}{2m} & i\in G_{1}^{+},\\ \frac{m_{1}^{-}}{2m}-\frac{1}{2} & i\in G_{1}^{-}. \end{array}\right)$$

It is easy to check that the pooled standard deviation is also independent of the effect size. As a result, the testing power with rank normalization converges to a constant strictly less than 1.0 as the effect size increases. More details can be found in Section 5 in the Additional file 1.

### Simulation studies

Extensive simulations are conducted to verify above theoretical predictions. We document these simulation studies in this section.

#### Simulation data

Two sets of simulated data are used in this study. Each set of data has two groups of *n *arrays representing gene expressions under two phenotypic groups (group *A* and group *B*). The numbers of up and down regulated genes are denoted by $m_{1}^{+}$ and *m*1-, respectively. Without loss of generality, group *B* is set to represent the normal phenotype, so up (down) regulation of a gene refers to its over (under) expression in group *A*. 

• **SIMU**: Each array has *m *= 1000 genes. The number of differentially expressed genes (DEGs) is set to be 100, which implies that the number of non-differentially expressed genes (NDEGs) is *m*_0 _= 900. For both groups, all genes are normally distributed with standard deviation *σ *= 0.35 which is estimated from the biological data. Every two distinct genes have correlation coefficient 0.9 which is estimated from the biological data. As a reference, the sample Pearson correlation coefficient averaged over all pairs of genes for biological data used in this study are: 0.91 for **HYPERDIP**, 0.93 for **TEL**, and 0.91 for **TALL**. The algorithm used to generate these correlated observations is stated in [36] and is similar to the method used in [37]. This high correlation between non-normalized gene expressions can introduce high correlation between the test statistics [38] and result in high instability of the list of DEGs. This phenomenon was documented and discussed in [39]. We also conduct simulations with non-homogeneous gene correlation structure and the results are similar to that of **SIMU**. Details can be found in Section 6 of the Additional file 1.

• The expectations of DEGs in group *A* ($y_{\text{ij}}^{A}$, *i *= 1, 2,…,*m*1+ + *m*1-, *j *= 1,2,…,*n*) are set to be a constant *e *for over-expressed genes (*i *= 1,…,*m*1+) and -*e *for under-expressed genes (*i*= *m*1- + 1,…, 100). Here the effect size *e *takes value in {0.2,0.4,⋯, 3.4,3.6}. $\left(m_{1}^{+},m_{1}^{-}\right)$ is set to be either (60,40) (balanced differential expression structure) or (90,10) (unbalanced differential expression structure). For all genes in group *B *and NDEGs in group *A*, their expectations are set to be 0. The sample size in each group is set to be *n*, taking values in {5,10}.

• **SIMU-BIO**: To match the statistical properties of real gene expression more closely and mimic other noise sources such as non-additive noise, we apply resampling method to the biological data to construct an additional set of data.

• We apply *t*-test to **HYPERDIP **and **TEL **(79 arrays chosen from each set) without any normalization procedure or multiple testing adjustment. At significance level 0.05, 734 genes are detected as DEGs with an unbalanced differential expression structure (677 up-regulated and 57 down-regulated). We record the mean difference across **HYPERDIP **and **TEL **for each DEG as its effect size (*e*_*i*_). Then we combine **HYPERDIP **and **TEL **data and randomly permute the arrays. After that we randomly choose 2*n *arrays and divide them into two groups *A *and *B *of *n *arrays each, mimicking two biological conditions without differentially expressed genes. Here the sample size *n *takes value in {5,10}. We add the recorded effect sizes to 734 genes (identified earlier) in group A. We also add addition effect size *e *to 677 up-regulated genes and -*e *to 57 down-regulated genes in group A where *e *takes value in {0,0.2,0.4, ⋯, 3.4,3.6}. These 734 genes are defined as our DEGs in this simulation. Similarly, we apply this resampling procedure to **TALL **and **TEL **(45 arrays chosen from each set) and 546 genes are defined to be DEGs with a balanced differential expression structure (259 up-regulated and 287 down-regulated). The sample size *n *takes value in {5,10} and the additional effect size *e *takes value in {0,0.2,0.4,⋯,3.4,3.6}.

### Hypothesis testing methods

We use Student’s *t*-test to compute unadjusted *p*-values and then apply the Bonferroni multiple testing adjustment to compute the adjusted *p*-values and control the family-wise error rate (FWER) at 0.05 level.

Two alternative tests, namely the Wilcoxon rank-sum test and permutation *N*-test are also used in this study. The results are largely consistent with those obtained from the *t*-test and can be found in Section 6 in the Additional file 1. The *N*-test is a multivariate nonparametric test which has been used to successfully select differentially expressed genes and gene combinations in microarray data analysis [23,40-42]. A brief introduction of this test can be found in Section 1 in the Additional file 1.

## Results and discussion

**Figure 3:**
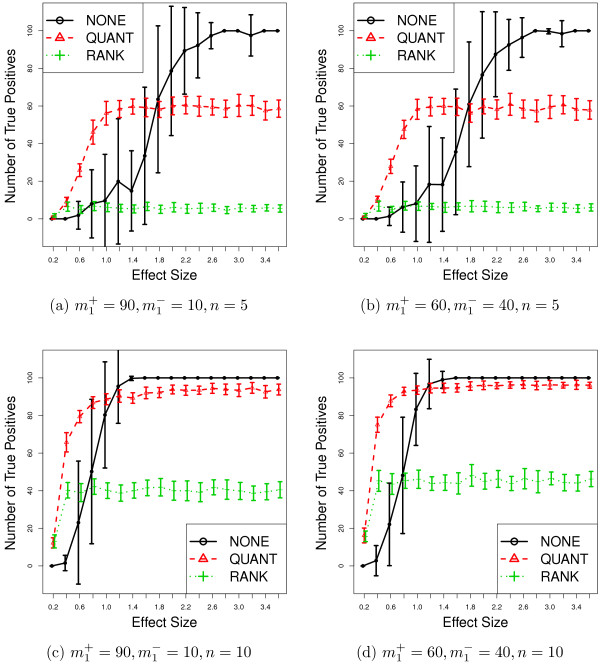
**Simulation results (SIMU). **Average number of true positives as functions of effect size for **SIMU**. The error bar represents one standard deviation above and below average. Total number of truly differentially expressed genes is 100 with *m*1+ up-regulated and *m*1- down-regulated genes, respectively. Data replicates: 20.

**Figure 4:**
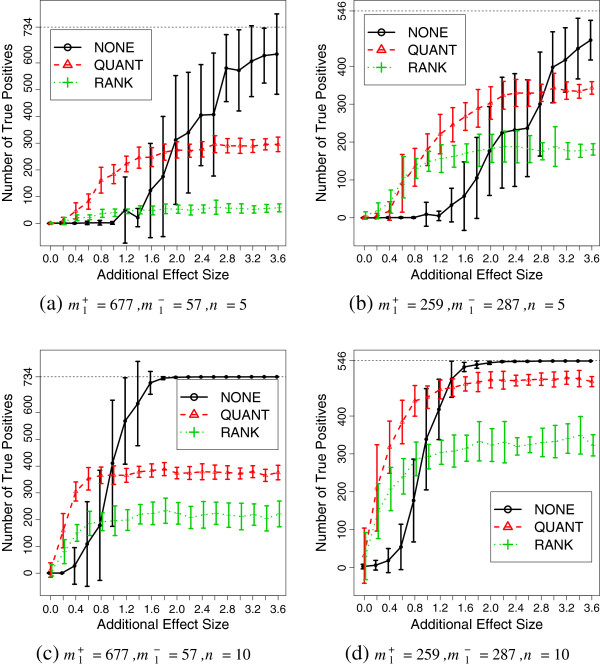
**Simulation results (SIMU-BIO). **Average number of true positives as functions of effect size for **SIMU-BIO**. The error bar represents one standard deviation above and below average. Total number of truly differentially expressed genes is $m_{1}^{+}+m_{1}^{1}$ with *m*1+ up-regulated and *m*1- down-regulated genes, respectively. Data replicates: 20.

We randomly generate 20 sets of data per tuning parameter for **SIMU **and **SIMU-BIO**. We apply normalization procedures first and then conduct hypothesis tests to obtain raw *p*-values. After that, we apply the Bonferroni multiple testing adjustment to get adjusted *p*-values. We declare a gene to be differentially expressed if its adjusted *p*-value is less than a prespecified significance level 0.05. The estimated mean and standard deviation of the true positives are reported in Figures 3 and 4. Various results with additional tests (Wilcoxon rank-sum test and permutation *N*-test), sample sizes (*n *= 15,20) and non-homogeneous gene correlation structure including false positive plots can be found in Section 6 in the Additional file 1.

By removing the noise from the observed gene expressions, quantile and rank normalization procedures improve the statistical power of the subsequent differential expression analyses when effect size is small. However, when *e *becomes large, the testing powers based on the normalized expressions converge to fixed numbers strictly less than 1.0. This confirms our previous theoretical derivations.

## Conclusions

Microarray technology has been used in many areas of biomedical research. Biomedical researchers rely on this technology to identify differentially expressed genes. Due to the “large *p*, small *n*” nature of the microarray data, multiple testing correction must be applied in differentially expression analysis. As we all know, stringent control of Type I error invariably comes with the price of reduced testing power. However, the success of most microarray studies depends critically on the ability of differential expression analysis to identify the “right genes” and researchers cannot afford to miss many these targets.

High statistical power can be achieved in a study with the following properties. 

1. An adequate sample size. Clearly, this is a reliable way to increase statistical power. Everyone seems to agree on it but not everyone practices it. Many years ago this was due to the high cost of conducting microarray experiments. Currently it only costs a fraction to obtain the same number of arrays. In a sense, the myth that “five arrays per group should be good enough” only reflects the fact that it takes a long time to change old, perhaps even anachronic habits.

2. Small variance. It is well known that a large proportion of the variance of gene expression is induced by undesirable systematic variations and various technical noise. Microarray technology has been evolving very fast in the past years and we think it is not unreasonable to assume that the technical noise level is getting lower. However, variance induced by biological heterogeneity will not be affected by the advances of technology. For certain data, using a normalization procedure, such as **QUANT **or **RANK**, can reduce this variance and help detect DEGs. We must point out that these elegant variance reduction procedures can also alter the mean expression and *increase *sample variance when the true effect size is large. This bias-variance trade-off is common in different branches of statistics and should not be conveniently ignored.

3. Strong true effect size. Based on our experience, this is often invoked as a reason to justify the use of small sample size in a study *a priori*. In our study, we demonstrate that one cannot simply “trade” sample size by effect size. Both our theoretical derivations and simulation studies indicate that as long as the sample size is small, the testing power of a typical gene differential expression analysis based on quantile or rank normalized data never reaches 100% no matter how large the effect size is. A large *n* is still critical for finding informative genes in this situation.

One main motivation of our study is to dismiss the dangerous idea that “five arrays per-group ought to be good enough for my study”. Our somewhat counter-intuitive findings suggest that if data with dramatic gene differentiation have only limited sample size (*e.g.*, less than 10 per group), rank and quantile normalizations may not be able to improve testing power as one expects. For such a scenario we recommend conducting an additional differential expression analysis with other normalization procedure or even without normalization first, and then compare/combine the results with the original analysis with quantile or rank normalization.

Although we choose to focus on the Affymetrix GeneChip platform throughout this paper, we believe our conclusions should be valid for other array platforms which require/recommend normalization, such as Affymetrix exon arrays, Illumina BeadChip arrays and many others. We hope this study can help biological researchers choose an appropriate normalization procedure in their experiments or even develop novel normalization procedures with better downstream testing power when the gene differential expression is dramatic.

## Competing interests

The authors declare that they have no competing interests.

## Authors’ contributions

All three authors have equal contribution to this paper including the original idea, study design, theoretical derivations, simulations and summary of the findings. All authors read and approved the final manuscript.

## Supplementary Material

Additional file 1 Supplementary material. Click here for file
