# Supplementary material for: The impact of quantile and rank normalization procedures on the testing power of gene differential expression analysis
Source: BMC Bioinformatics. 2013 Apr 11;14:124. doi: 10.1186/1471-2105-14-124 (PMC3660216; doi:10.1186/1471-2105-14-124)
Supplement: Additional file 1 — Supplementary material. [file 1471-2105-14-124-S1.pdf]

Supplementary Material: The Impact of Quantile and Rank Normalization Procedures on Testing Power in Differential Gene Expression Analysis

Xing Qiu Hulin Wu Rui Hu*

1 The N-statistic

We choose a multivariate nonparametric N-distance with the Euclidean kernel as a measure of the distance between two multivariate probability distributions. Using the same notation as in the main text, the sample N-distance across conditions A and B for gene i is defined as follows:

$$N_i = \frac{2}{n^2}\sum_{k=1}^{n}\sum_{l=1}^{n} L(x_{ik}^A, x_{il}^B) - \frac{1}{n^2}\sum_{k=1}^{n}\sum_{l=1}^{n} L(x_{ik}^A, x_{il}^A) - \frac{1}{n^2}\sum_{k=1}^{n}\sum_{l=1}^{n} L(x_{ik}^B, x_{il}^B),$$

where $L(x, y) = |x - y|$ is the kernel defined by the Euclidean distance. We apply the following algorithm to calculate the permutation-based p-values.

1. Randomly shuffle the arrays in two different conditions, then split them into two groups of equal size.
2. Compute the N-statistic for each gene.
3. Repeat the above steps for $K = 1,000$ times, record the permutation based N-statistics as $N_{ik}, i = 1, \ldots, m,\ k = 1, \ldots, K$. They can be used to construct the permutation based null distribution for each index i.
4. Compute N_i, the N-statistic for each gene without random shuffles.
5. Obtain the permutation based p-value, p_i, by comparing N_i with the null distribution constructed from N_{ik}. Specifically, p_i is defined to be $\frac{\#(N_{ik} \geqslant N_i)}{K}$, the proportion of N_{ik} which is greater than or equal to N_i.

2 A graphical illustration of the bias induced by quantile normalization

Denote the reference quantile array constructed from one group by $\mathbf{q}^c$, $c = A, B$, we have

$$q_r = \frac{q_r^A + q_r^B}{2}, \quad q_r^c = \frac{1}{n}\sum_{k=1}^{n} y_{(r),k}^c,\ c = A, B. \tag{1}$$

In other words, the reference array $\mathbf{q}$ is computed by averaging both DEGs and NDEGs over arrays in two phenotypic groups, so the m_1^+ over-expressed genes and m_1^- under-expressed genes in group A are "mixed up" with NDEGs of the same rank from group B.

Figure 1 shows the empirical density functions of $\mathbf{q}^A$, $\mathbf{q}^B$, and $\mathbf{q}$ computed by pooling q_r^c, $r = 1, 2, \ldots, 1000$, from 200 repetitions of **SIMU**. Strictly speaking, these are density estimates from random samples q_r^c according to the discrete uniform distribution on $\{1, 2, \ldots, 1000\}$. Since none of the genes in group B are under/over-expressed, $\mathbf{q}^B$ roughly follows a normal distribution (Figure 1(b)). The right, left and middle components of Figure 1(a) represent the top m_1^+, bottom m_1^- and the rest m_0 empirical quantiles in group

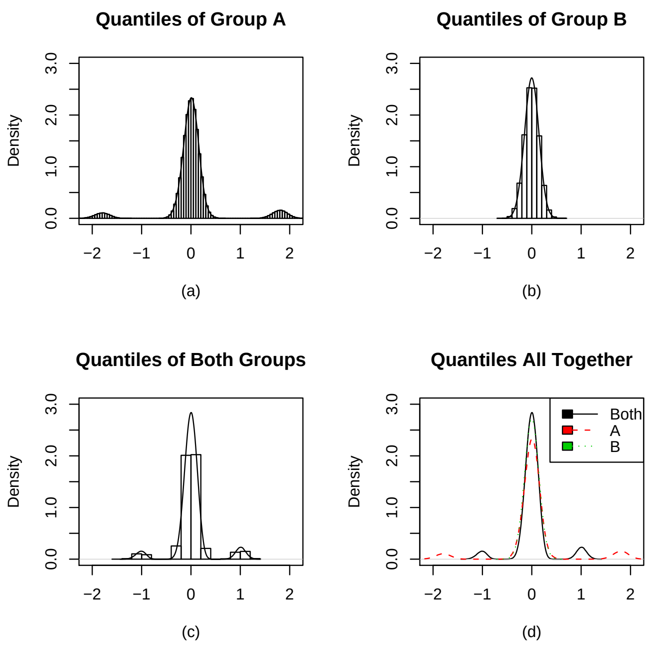

Figure 1: Empirical density estimates of the quantile reference arrays. Total number of genes is $m = 1000$. Total number of truly differentially expressed genes is $m_1^+ = 100$. The sample size is $n = 10$ and the true effect size is $e^+ = -e^- = 1.8$. Estimates are based on 200 repetitions. (a) $\mathbf{q}^A$. (b) $\mathbf{q}^B$. (c) $\mathbf{q}$. (d) Three quantile density estimates in one plot.

A, respectively. Since $e^+ = -e^- = 1.8$ is much larger than the standard deviation $\sigma = 0.35$, the over and under-expressed genes in group A almost always take up the top m_1 and bottom m_2 places, respectively, so these three components are well separated. Due to reasons elaborated in Section 3.1 of the main text, the center of the right (left) component of Figure 1(c), which represents the top m_1^+ (bottom m_1^-) q_r, is closer to the middle component than it is in Figure 1(a). It explains that **QUANT** can decrease the effect size for DEGs.

3 Growth of sample mean difference and pooled sample standard deviation as functions of effect size

In group A, over(under)-expressed genes tend to have high (low) ranks in each array. When the effect size is large, the DEGs in group A effectively take up all the top and bottom ranks, so the NDEGs in group A can only compete for ranks between $m_1^- + 1$ and $m - m_1^+$. We assume that the m_1^+ up-regulated genes almost always take the top m_1^+ ranks with equal chances and the m_1^- down-regulated genes almost always take the bottom m_1^- ranks with equal chances. By using conditional expectation, we can compute that for $i \in G_1^+$,

$$\begin{aligned}
\mathrm{E}(y_{i\cdot}^{*A}) &\approx \frac{1}{m_1^+} \sum_{r=m_1^-+m_0+1}^{m} \mathrm{E}\left(q_r\right) \\
&\approx \frac{1}{2m_1^+ n} \left(\sum_{k=1}^{n} \sum_{j \in G_1^+} \mathrm{E}(y_{jk}^A) + \sum_{l=1}^{n} \sum_{r=m_1^-+m_0+1}^{m} \mathrm{E}(y_{(r),l}^B) \right) \\
&= \frac{e^+ + \delta_1^+}{2}.
\end{aligned}$$

where $\delta_1^+ = \frac{1}{m_1^+}\sum_{r=m_1^-+m_0+1}^{m} \mathrm{E}(y_{(r),\cdot}^B)$ represents the mean expectation of the top m_1^+ ordered expression levels provided that there is no differentially expressed gene. Similarly,

$$\mathrm{E}(y_{i\cdot}^{*A}) \approx \frac{e^- + \delta_1^-}{2}, \quad i \in G_1^-,$$

where $\delta_1^- = \frac{1}{m_1^-}\sum_{r=1}^{m_1^-} \mathrm{E}(y_{(r),\cdot}^B)$.

Based on Equation (1) in the main text, mean gene expressions in group B are all the same. We further assume that all genes in group B have equal chances to take (array-specific) ranks from 1 to m. Then,

$$\begin{aligned}&\mathrm{E}(y_{i\cdot}^{*B}|r_{i\cdot}^B)\\ &\approx \begin{cases} \frac{e^+ + \delta_1^+}{2} \text{ with probability } \frac{m_1^+}{m} \ \ (r_{i\cdot}^B \in \text{top } m_1^+), \\ \frac{e^- + \delta_1^-}{2} \text{ with probability } \frac{m_1^-}{m} \ \ (r_{i\cdot}^B \in \text{bottom } m_1^-), \\ \frac{\delta_0}{2} \text{ with probability } \frac{m_0}{m} \ \ (r_{i\cdot}^B \in \text{middle } m_0), \end{cases}\end{aligned}$$

where $\delta_0 = \frac{1}{m_0}\sum_{r=m_1^-+1}^{m_1^-+m_0} \mathrm{E}(y_{(r),\cdot}^B)$ and

$$m_1^+\delta_1^+ + m_1^-\delta_1^- + m_0\delta_0 = \sum_{j=1}^{m} \mathrm{E}(y_{j\cdot}^B) = 0.$$

Compared with (1) in the main text, the expected expressions of quantile normalized DEGs have been altered by **QUANT**. According to definition, all δ_1^+, δ_1^- and δ_0 do not depend on e^+ and e^-. So they can be ignored for large e^+ and e^-. Consequently if the effect sizes are very large, **QUANT** reduces them by about 50%. A graphical illustration of this bias can be found in Section 2.

Based on the above calculations, the expected sample differences for up-regulated DEGs ($i \in G_1^+$) are

$$\begin{aligned}&\mathrm{E}(y_{i\cdot}^{*A} - y_{i\cdot}^{*B}|r_{i\cdot}^B)\\ &\approx \begin{cases} 0, \text{ with probability } \frac{m_1^+}{m} \ \ (r_{i\cdot}^B \in \text{top } m_1^+), \\ \frac{e^+ + \delta_1^+}{2} - \frac{e^- + \delta_1^-}{2}, \text{ with probability } \frac{m_1^-}{m} \ \ (r_{i\cdot}^B \in \text{bottom } m_1^-), \\ \frac{e^+ + \delta_1^+}{2} - \frac{\delta_0}{2}, \text{ with probability } \frac{m_0}{m} \ \ (r_{i\cdot}^B \in \text{middle } m_0). \end{cases}\end{aligned}$$

So the expected sample mean differences for these normalized DEGs are

$$\begin{aligned}&\mathrm{E}(\bar{y}_{i\cdot}^{*A} - \bar{y}_{i\cdot}^{*B}|r_{i1}^B, \cdots, r_{in}^B)\\ &\propto \begin{cases} O(1), \text{ with probability } (\frac{m_1^+}{m})^n \ \ (\text{all } r_{i\cdot}^B \in \text{top } m_1^+), \\ O(e^+, e^-), \text{ with probability } 1 - (\frac{m_1^+}{m})^n \\ \qquad\qquad (\text{some } r_{i\cdot}^B \notin \text{top } m_1^+). \end{cases}\end{aligned} \tag{2}$$

Similarly for down-regulated DEGs ($i \in G_1^-$),

$$\begin{aligned}&\mathrm{E}(\bar{y}_{i\cdot}^{*A} - \bar{y}_{i\cdot}^{*B}|r_{i1}^B, \cdots, r_{in}^B)\\ &\propto \begin{cases} O(1), \text{ with probability } (\frac{m_1^-}{m})^n \ \ (\text{all } r_{i\cdot}^B \in \text{bottom } m_1^-), \\ O(e^+, e^-), \text{ with probability } 1 - (\frac{m_1^-}{m})^n \\ \qquad\qquad (\text{some } r_{i\cdot}^B \notin \text{bottom } m_1^-). \end{cases}\end{aligned} \tag{3}$$

On the other hand, $\hat{\sigma}_i^*$, the pooled sample standard deviation, grows at most linearly as a function of e^+ and e^-. To see this, we check $\hat{\sigma}_i^{*A}$ and $\hat{\sigma}_i^{*B}$ separately.

For up-regulated genes ($i \in G_1^+$),

$$
\begin{aligned}
(\hat{\sigma}_i^{*A})^2 &\propto \sum_{j=1}^{n}(y_{ij}^{*A}-\bar{y}_{i\cdot}^{*A})^2 \propto \sum_{j=1}^{n}\left(\sum_{k=1}^{n}(y_{ij}^{*A}-y_{ik}^{*A})\right)^2 \\
&= \sum_{j=1}^{n}\left(\sum_{k=1}^{n}\left(\sum_{l=1}^{n} y^A_{(r^A_{ij}),l}+\sum_{l=1}^{n} y^B_{(r^A_{ij}),l}-\sum_{l=1}^{n} y^A_{(r^A_{ik}),l}-\sum_{l=1}^{n} y^B_{(r^A_{ik}),l}\right)\right)^2 \\
&= \sum_{j=1}^{n}\left(\sum_{k=1}^{n}\sum_{l=1}^{n}\left((y^A_{(r^A_{ij}),l}-e^+)-(y^A_{(r^A_{ik}),l}-e^+)\right)+\sum_{k=1}^{n}\sum_{l=1}^{n}\left(y^B_{(r^A_{ij}),l}-y^B_{(r^A_{ik}),l}\right)\right)^2
\end{aligned}
\tag{4}
$$

according to Equation (5) in the main text. If e^+ is large enough, the over-expressed DEGs in group A almost always take up the top m_1^+ places, so $m_1^- + m_0 + 1 \leqslant r_{ij}^A \leqslant m$ and $m_1^- + m_0 + 1 \leqslant r_{ik}^A \leqslant m$. Then, $y^A_{(r^A_{ij}),l}$ and $y^A_{(r^A_{ik}),l}$ are two of the m_1^+ non-normalized over-expressed DEG expressions in sample l, both approximately having expectations e^+. Consequently, $(y^A_{(r^A_{ij}),l}-e^+)$, $(y^A_{(r^A_{ik}),l}-e^+)$, $y^B_{(r^A_{ij}),l}$, and $y^B_{(r^A_{ik}),l}$ are all independent of e^+. Therefore, $(\hat{\sigma}_i^{*A})^2$ does not depend on e^+ for $i \in G_1^+$. Similarly, $(\hat{\sigma}_i^{*A})^2$ does not depend on e^- for down regulated genes ($i \in G_1^-$).

On the other hand, for gene expressions in group B,

$$
\begin{aligned}
(\hat{\sigma}_i^{*B})^2 &\propto \sum_{j=1}^{n}\left(\sum_{k=1}^{n}(y_{ij}^{*B}-y_{ik}^{*B})\right)^2 \\
&= \sum_{j=1}^{n}\left(\sum_{k=1}^{n}\sum_{l=1}^{n}\left(y^A_{(r^B_{ij}),l}-y^A_{(r^B_{ik}),l}\right)+\sum_{k=1}^{n}\sum_{l=1}^{n}\left(y^B_{(r^B_{ij}),l}-y^B_{(r^B_{ik}),l}\right)\right)^2.
\end{aligned}
\tag{5}
$$

Again we assume that each r_{ij}^B, $j = 1, \ldots, n$, has equal probability to take value from 1 to m. So r_{ij}^B has $\frac{m_1^+}{m}$ probability to take value from one of the top ranks, $\{m_1^- + m_0 + 1, \ldots, m\}$; $\frac{m_1^-}{m}$ probability to take value from one of the bottom ranks, $\{1, \ldots, m_1^-\}$ (down regulated genes); $\frac{m_0}{m}$ probability to take value from the medium ranks (NDEGs). Since n samples are independent of each other, the probability for all $\{r_{i1}^B, \ldots, r_{in}^B\}$ to take values in $\{m_1^- + m_0 + 1, \ldots, m\}$ is $(\frac{m_1^+}{m})^n$. In such a situation, $y^A_{(r^B_{ij}),l} - y^A_{(r^B_{ik}),l} = (y^A_{(r^B_{ij}),l} - e^+) - (y^A_{(r^B_{ik}),l} - e^+)$ does not depend on e^+ since $y^A_{(r^B_{ij}),l}$ and $y^A_{(r^B_{ik}),l}$ are two of the m_1^+ non-normalized over-expressed DEG expressions in sample l, both approximately having expectations e^+. Similarly, the probability for all $\{r_{i1}^B, \ldots, r_{in}^B\}$ to take values in $\{1, \ldots, m_1^-\}$ is $(\frac{m_1^-}{m})^n$ and $y^A_{(r^B_{ij}),l} - y^A_{(r^B_{ik}),l}$ does not depend on e^- in such a situation. Also, the probability for all $\{r_{i1}^B, \ldots, r_{in}^B\}$ to take values in $\{m_1^- + 1, \ldots, m_1^- + m_0\}$ is $(\frac{m_0}{m})^n$. In this situation, $y^A_{(r^B_{ij}),l} - y^A_{(r^B_{ik}),l}$ does not depend on e^+ or e^- either since $y^A_{(r^B_{ij}),l}$ and $y^A_{(r^B_{ik}),l}$ are two of the m_0 non-normalized NDEG expressions in sample l, both approximately having expectations 0. Except for these three cases, $y^A_{(r^B_{ij}),l} - y^A_{(r^B_{ik}),l} \propto e^+$ or e^- since $y^A_{(r^B_{ij}),l}$ and $y^A_{(r^B_{ik}),l}$ belong to two of three groups in sample l, i.e., m_0 non-normalized NDEG expressions which approximately have expectation 0, m_1^+ non-normalized over-expressed DEG expressions which approximately have expectation e^+ and m_1^- non-normalized under-expressed DEG expressions which approximately have expectation e^-. Also noticing that $y^B_{(r^B_{ij}),l} - y^B_{(r^B_{ik}),l}$ does not depend on e^+ or e^-, we have

$$
\begin{aligned}
&\mathrm{E}\left((\hat{\sigma}_i^{*B})^2 | r_{i1}^B, \cdots, r_{in}^B\right) \\
&\propto \begin{cases} O(1), \text{ all } r_{i\cdot}^B \in \text{top } m_1^+, \text{ or middle } m_0, \text{ or bottom } m_1^- \text{ with probability } (\frac{m_0}{m})^n + (\frac{m_1^+}{m})^n + (\frac{m_1^-}{m})^n \\ O((e^+)^2, (e^-)^2), \text{ otherwise with probability } 1 - (\frac{m_0}{m})^n - (\frac{m_1^+}{m})^n - (\frac{m_1^-}{m})^n. \end{cases}
\end{aligned}
\tag{6}
$$

Thus,

$$\mathrm{E}(\hat{\sigma}_i^{*B})^2 \propto \left(\left(\frac{m_0}{m}\right)^n + \left(\frac{m_1^+}{m}\right)^n + \left(\frac{m_1^-}{m}\right)^n\right) O(1) + \left(1 - \left(\frac{m_0}{m}\right)^n - \left(\frac{m_1^+}{m}\right)^n - \left(\frac{m_1^-}{m}\right)^n\right) O((e^+)^2, (e^-)^2). \tag{7}$$

So $(\hat{\sigma}_i^{*B})^2$ grows at most quadratically as a function of $(e^+)^2$ and $(e^-)^2$. Therefore, $\hat{\sigma}_i^*$, the pooled sample deviation, grows at most *linearly* w.r.t. e^+ and e^-.

4 Convergence of doubly noncentral t-distribution

Suppose statistic T follows a doubly noncentral t-distribution with ν degrees of freedom and noncentrality parameters (γ, λ) [1]. Here γ is the numerator noncentrality parameter and λ is the denominator noncentrality parameter (from noncentral χ^2). Symbolically,

$$T = \frac{U + \gamma}{\chi_\nu(\lambda)/\sqrt{\nu}}, \tag{8}$$

where U follows standard normal distribution and $\chi_\nu(\lambda)$ follows noncentral χ^2 distribution with ν degrees of freedom and noncentrality parameter λ. It is known that the rth moment of T is [1]

$$\mathrm{E}(T^r) = (\frac{\nu}{2})^{\frac{r}{2}} \mathrm{E}[(U+\gamma)^r] \frac{\Gamma((\nu - r)/2)}{\Gamma(\nu/2)} M(\frac{r}{2}; \frac{\nu}{2}; -\frac{\lambda}{2}), \tag{9}$$

where $\Gamma(\cdot)$ is the gamma function and $M(\cdot;\cdot;\cdot)$ is the confluent hypergeometric function. From [2] we know

$$M(a;b;z) \propto \frac{\Gamma(b)}{\Gamma(b-a)} (-z)^{-a} \sum_{i=0}^{\infty} \frac{\Gamma(a+i)\Gamma(a-b-1+i)}{\Gamma(a)\Gamma(a-b-1) i! (-z)^i}.$$

Therefore,

$$\mathrm{E}(T^r) \propto (\frac{\nu}{2})^{\frac{r}{2}} \mathrm{E}[(U+\gamma)^r] (\frac{\lambda}{2})^{-\frac{r}{2}} \sum_{i=0}^{\infty} \frac{\Gamma(\frac{r}{2}+i)\Gamma(\frac{r-\nu}{2}-1+i)}{\Gamma(\frac{r}{2})\Gamma(\frac{r-\nu}{2}-1) i! (\frac{\lambda}{2})^i}. \tag{10}$$

Specially, if $\lambda = \gamma^2$,

$$\mathrm{E}(T^r) \propto (\nu)^{\frac{r}{2}} \frac{\mathrm{E}[(U+\gamma)^r]}{\gamma^r} \sum_{i=0}^{\infty} \frac{\Gamma(\frac{r}{2}+i)\Gamma(\frac{r-\nu}{2}-1+i)}{\Gamma(\frac{r}{2})\Gamma(\frac{r-\nu}{2}-1) i! (\frac{\gamma^2}{2})^i}. \tag{11}$$

Noticing $\mathrm{E}[(U+\gamma)^r] \propto \gamma^r$, we have $\mathrm{E}(T^r) < \infty$ as $\gamma \to \infty$. Consequently, the doubly noncentral t-distribution converges to a distribution with finite all order moments when γ approaches infinity.

5 Convergence Results Related to the Rank Normalization

Based on the assumptions made in the main text, it is clear that when the effect size e^+ and $|e^-|$ are large, the over-expressed genes always take up the top m_1^+ ranks and the under-expressed genes always take up the bottom m_1^- ranks in group A. Consequently, the rank normalized expressions, $y_{ij}^{*c} = \frac{r_{ij}^{*c}}{m}$, approximately have the following uniform distribution:

$$y_{ij}^{*c} \sim \begin{cases} U(1 - \frac{m_1^+}{m}, 1), & c = A,\ i \in G_1^+, \\ U(0, \frac{m_1^-}{m}), & c = A,\ i \in G_1^-, \\ U(\frac{m_1^-}{m}, 1 - \frac{m_1^+}{m}), & c = A,\ i \in G_0, \\ U(0,1), & c = B. \end{cases} \tag{12}$$

Therefore, the normalized gene expressions do not depend on the effect size anymore. the expected group differences for rank normalized DEGs are

$$\mathrm{E}\left(y_{i\cdot}^{*A} - y_{i\cdot}^{*B}\right) \approx \begin{cases} \frac{1}{2} - \frac{m_1^+}{2m} & i \in G_1^+, \\ \frac{m_1^-}{2m} - \frac{1}{2} & i \in G_1^-. \end{cases} \tag{13}$$

the expectation of pooled variances are

$$\mathrm{E}\left(\frac{\left(\hat{\sigma}_i^{*A}\right)^2 + \left(\hat{\sigma}_i^{*B}\right)^2}{2}\right) \approx \frac{\left(\sigma_i^{*A}\right)^2 + \left(\sigma_i^{*B}\right)^2}{2} \approx \begin{cases} \frac{1}{24}\left(\frac{m_1^+}{m}\right)^2 + \frac{1}{24} & i \in G_1^+, \\ \frac{1}{24}\left(\frac{m_1^-}{m}\right)^2 + \frac{1}{24} & i \in G_1^-. \end{cases} \tag{14}$$

So the two sample t-statistics have the following approximation independent of the effect size

$$\mathrm{E}t_i^* \approx \sqrt{\frac{n}{2}} \cdot \frac{\mathrm{E}\left(y_{i\cdot}^{*A} - y_{i\cdot}^{*B}\right)}{\sqrt{\mathrm{E}\left(\frac{\left(\hat{\sigma}_i^{*A}\right)^2 + \left(\hat{\sigma}_i^{*B}\right)^2}{2}\right)}} \approx \begin{cases} \sqrt{3n} \cdot \frac{1 - \frac{m_1^+}{m}}{\sqrt{\left(\frac{m_1^+}{m}\right)^2 + 1}}, & i \in G_1^+, \\ \sqrt{3n} \cdot \frac{\frac{m_1^-}{m} - 1}{\sqrt{\left(\frac{m_1^-}{m}\right)^2 + 1}}, & i \in G_1^-. \end{cases} \tag{15}$$

As a result, the testing power with rank normalization converges to a constant strictly less than 1.0 as the effect size increases. This constant depends on n, the sample size, and $\frac{m_1^+}{m}$ and $\frac{m_1^-}{m}$, the *proportions* of up and down regulated genes.

6 More results of simulation studies

We simulate one more set of data **SIMU-RANDOMCORR** with non-homogeneous gene correlation structure.

- **SIMU-RANDOMCORR**: Each array has $m = 1000$ genes. The number of differentially expressed genes (DEGs) is set to be 100, which implies that the number of non-differentially expressed genes (NDEGs) is $m_0 = 900$. For both groups, all genes are normally distributed with standard deviation $\sigma = 0.35$ which is estimated from the biological data. Every two distinct genes have correlation coefficient ρ which is a random number from uniform distribution on $[0.1, 0.9]$.

  The expectations of DEGs in group A (y_{ij}^A, $i = 1, 2, \ldots, m_1^+ + m_1^-$, $j = 1, 2, \ldots, n$) are set to be a constant e for over-expressed genes ($i = 1, \ldots, m_1^+$) and $-e$ for under-expressed genes ($i = m_1^- + 1, \ldots, 100$). Here the effect size e takes value in $\{0.2, 0.4, \cdots, 3.4, 3.6\}$. (m_1^+, m_1^-) is set to be either $(60, 40)$ (balanced differential expression structure) or $(90, 10)$ (unbalanced differential expression structure). For all genes in group B and NDEGs in group A, their expectations are set to be 0. The sample size in each group is set to be n, taking values in $\{5, 10\}$.

The simulation results with t test, Wilcoxon rank sum test and N-test are presented in Figures 2, 3, 4, 5, 6, 7, 8, 9, 10, 11, 12, 13, 14 and 15. We choose a little bit larger sample size $n = 10$ and $n = 15$ for simulations from normal distribution (**SIMU**) and $n = 15$ and $n = 20$ for simulations from biological data (**SIMU-BIO**). The testing powers all converge to fixed numbers smaller than 1.0 when effect size becomes large regardless of hypothesis testing methods. If the sample size is large enough ($n = 20$), it is hard to see this phenomenon empirically without more repetitions since the real testing power is too close to 1. We may still be able to observe the testing power converging to a fixed number strictly less than 1.0 when conducting a simulation with more repetitions. Also, the type 1 errors in SIMU-BIO are not well controlled. This is due to the fact that the SIMU-BIO data come from the biological data permutation. We "define" the truly differentially expressed genes by t test based on non-normalized data, which may miss some truly differentially expressed genes. After quantile or rank normalizations, these "missed" genes may be identified.

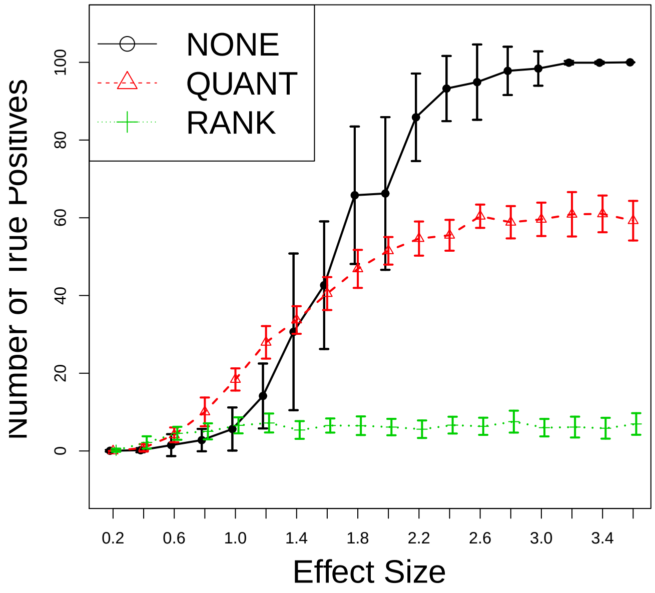

(a) $m_1^+ = 90, m_1^- = 10, n = 5$

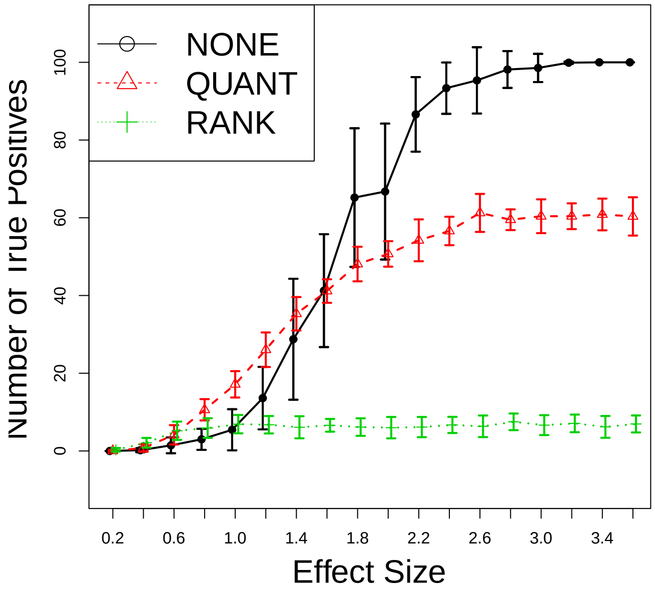

(b) $m_1^+ = 60, m_1^- = 40, n = 5$

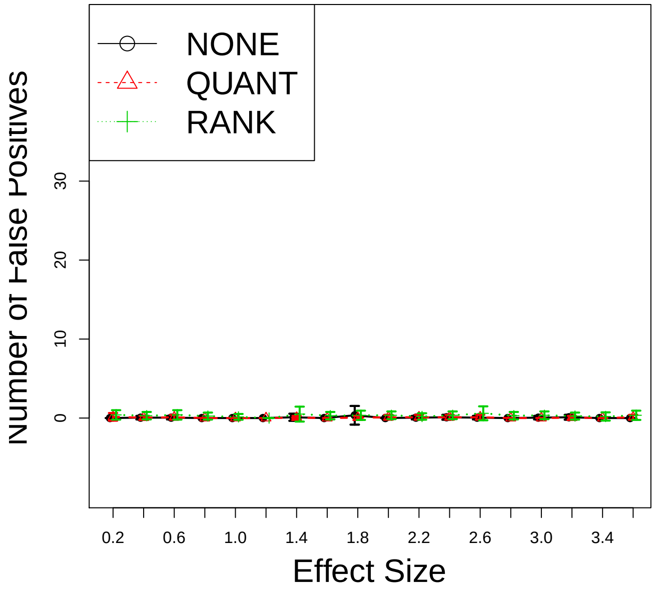

(c) $m_1^+ = 90, m_1^- = 10, n = 5$

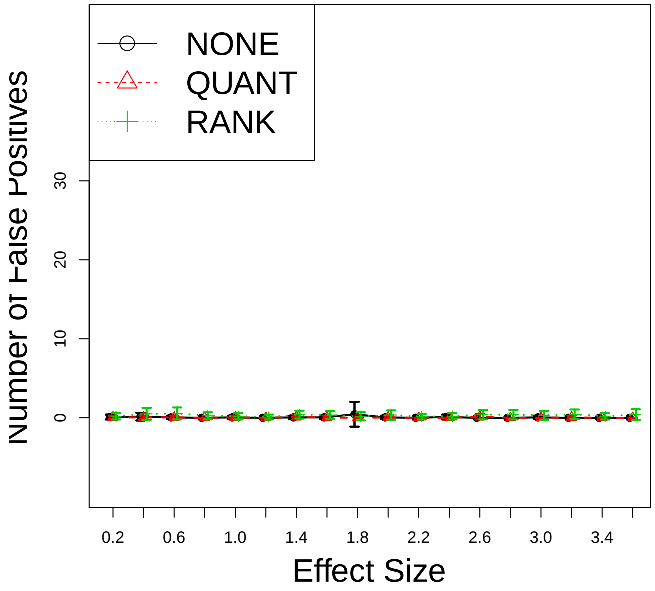

(d) $m_1^+ = 60, m_1^- = 40, n = 5$

Figure 2: Average number of true and false positives as functions of effect size for **SIMU-RANDOMCORR**. The error bar represents one standard deviation above and below average. Total number of truly differentially expressed genes is 100 with m_1^+ up-regulated and m_1^- down-regulated genes, respectively. DEGs are selected by t-test. Data replicates: 20.

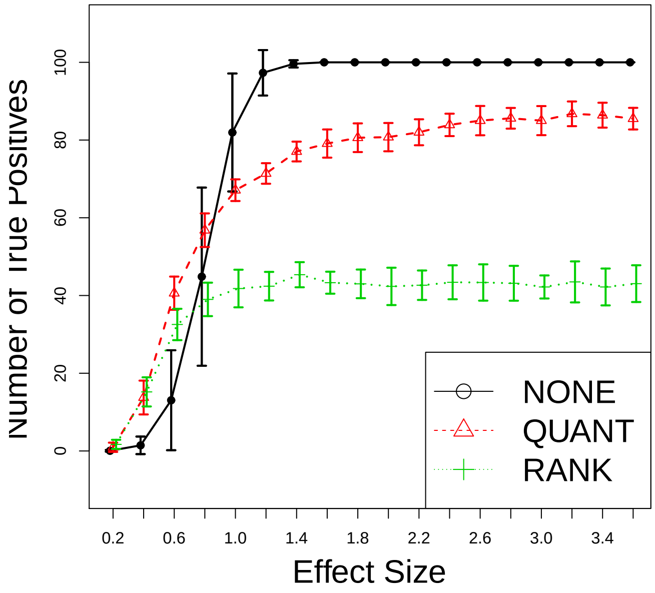

(a) $m_1^+ = 90, m_1^- = 10, n = 10$

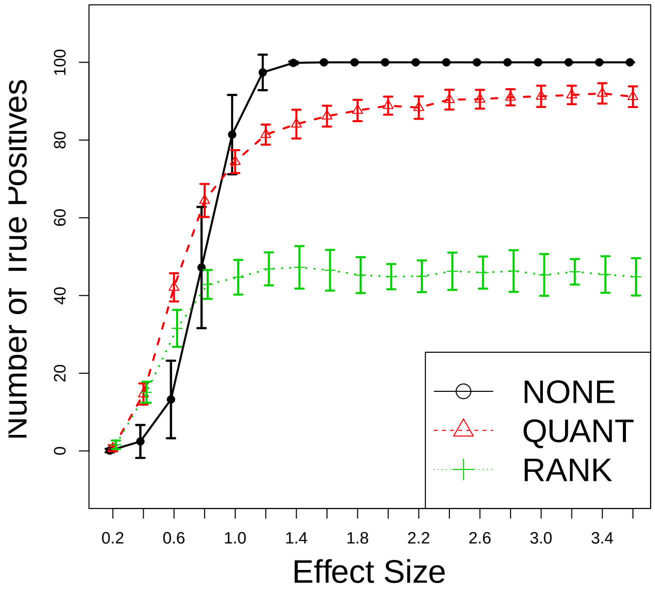

(b) $m_1^+ = 60, m_1^- = 40, n = 10$

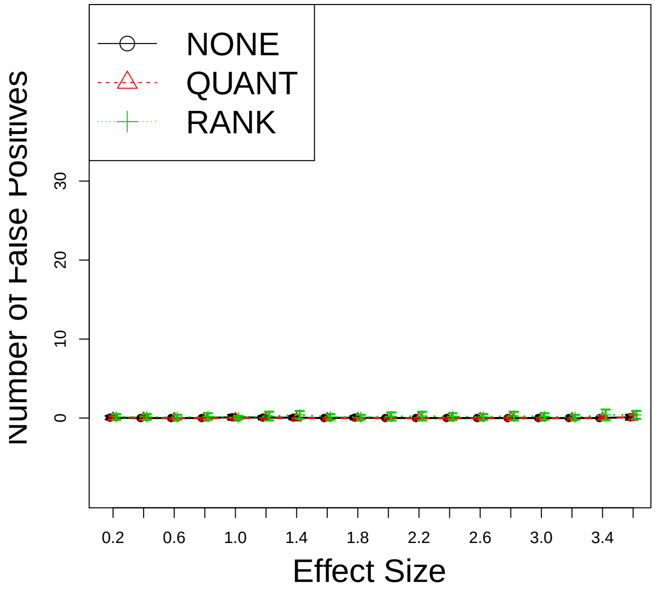

(c) $m_1^+ = 90, m_1^- = 10, n = 10$

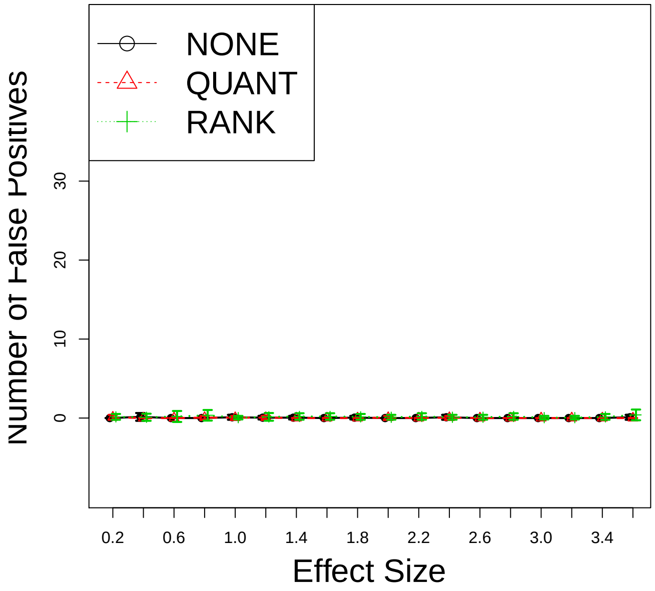

(d) $m_1^+ = 60, m_1^- = 40, n = 10$

Figure 3: Average number of true and false positives as functions of effect size for **SIMU-RANDOMCORR**. The error bar represents one standard deviation above and below average. Total number of truly differentially expressed genes is 100 with m_1^+ up-regulated and m_1^- down-regulated genes, respectively. DEGs are selected by t-test. Data replicates: 20.

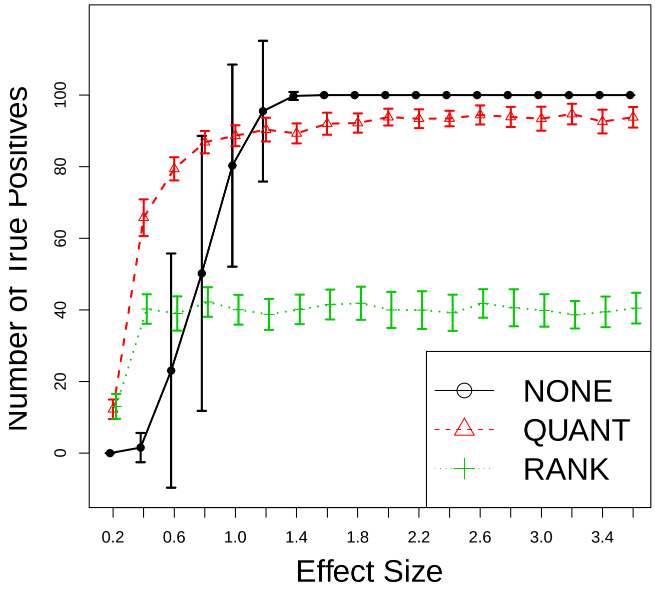

(a) $m_1^+ = 90, m_1^- = 10, n = 10$

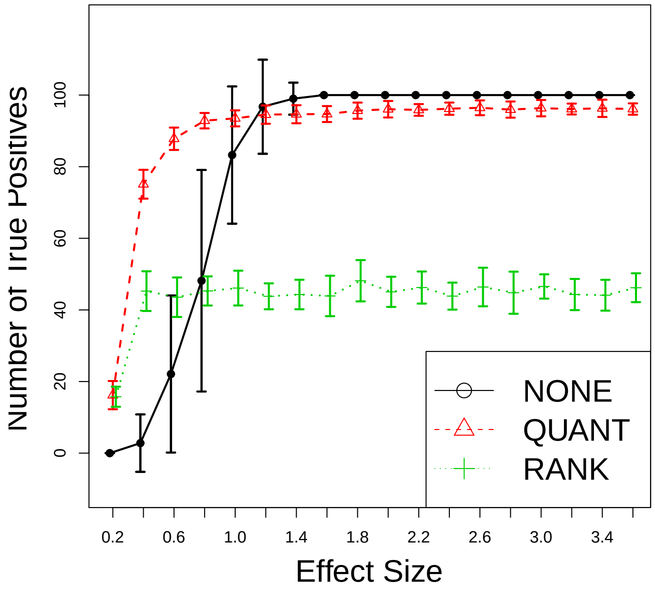

(b) $m_1^+ = 60, m_1^- = 40, n = 10$

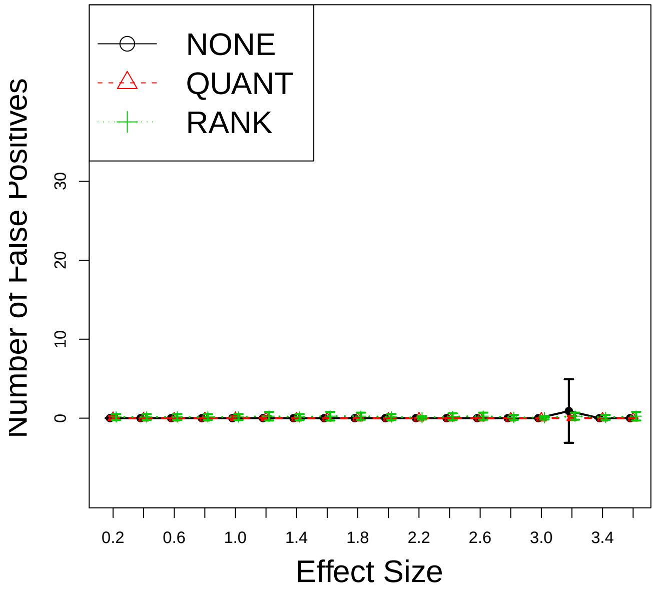

(c) $m_1^+ = 90, m_1^- = 10, n = 10$

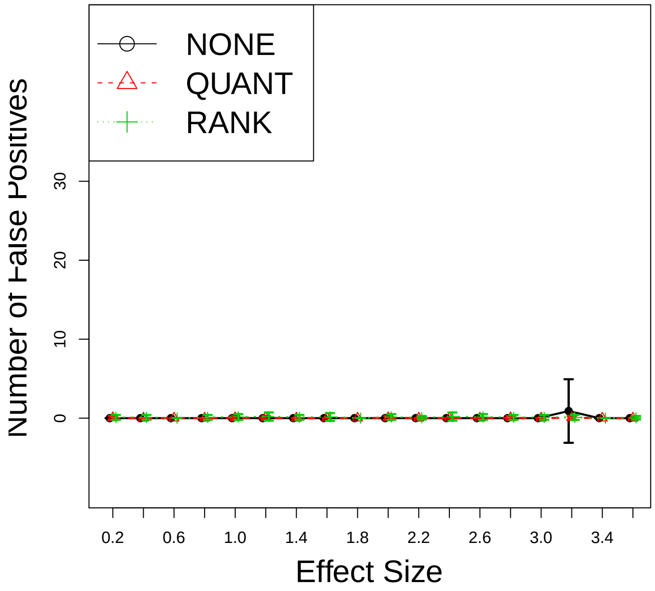

(d) $m_1^+ = 60, m_1^- = 40, n = 10$

Figure 4: Average number of true and false positives as functions of effect size for **SIMU**. The error bar represents one standard deviation above and below average. Total number of truly differentially expressed genes is 100 with m_1^+ up-regulated and m_1^- down-regulated genes, respectively. DEGs are selected by t-test. Data replicates: 20.

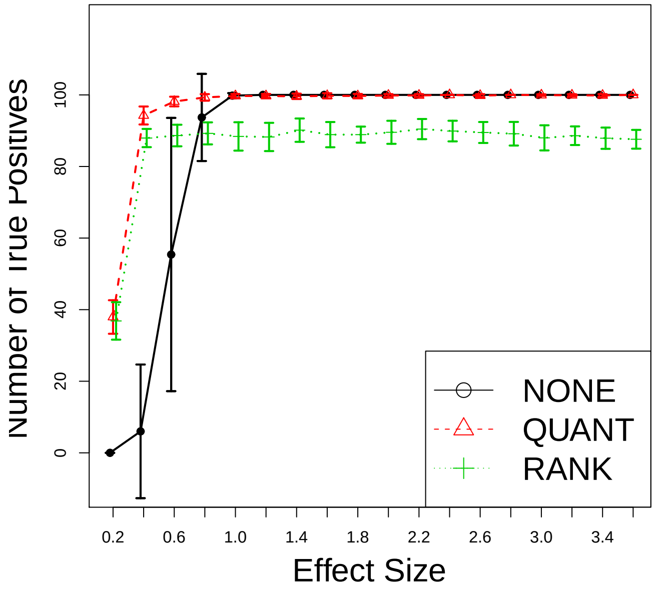

(a) $m_1^+ = 90, m_1^- = 10, n = 15$

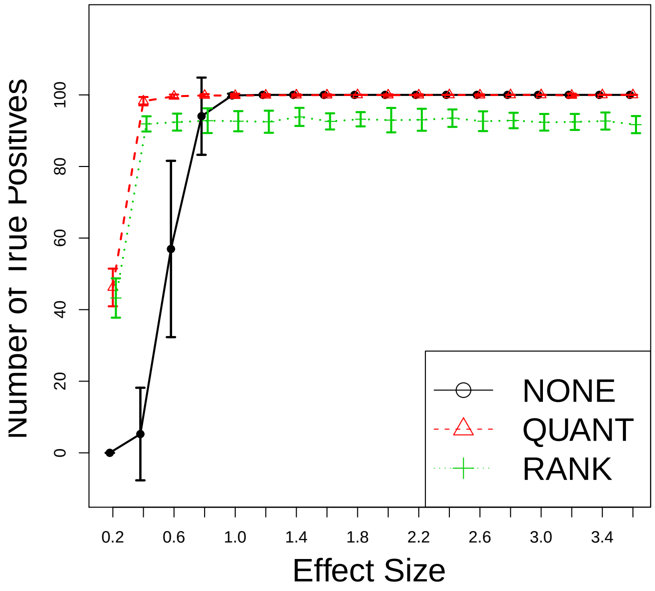

(b) $m_1^+ = 60, m_1^- = 40, n = 15$

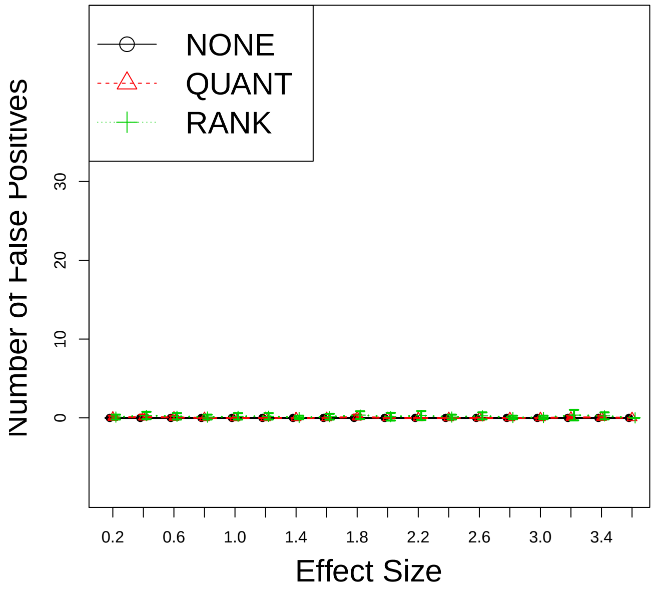

(c) $m_1^+ = 90, m_1^- = 10, n = 15$

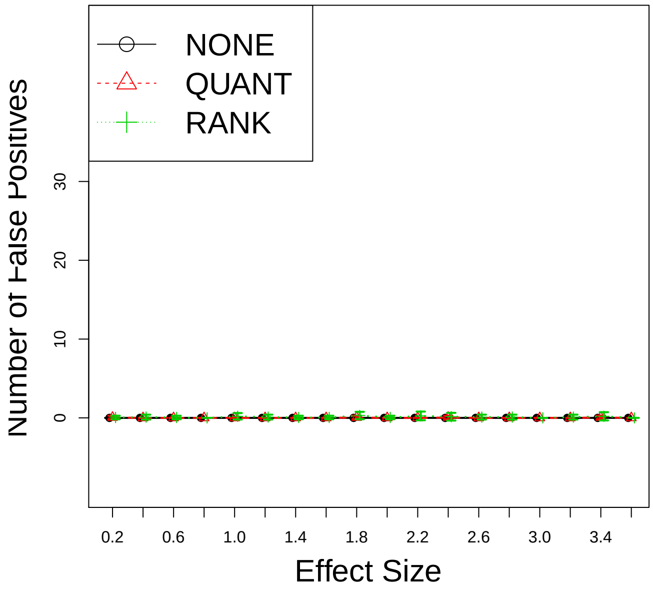

(d) $m_1^+ = 60, m_1^- = 40, n = 15$

Figure 5: Average number of true and false positives as functions of effect size for **SIMU**. The error bar represents one standard deviation above and below average. Total number of truly differentially expressed genes is 100 with m_1^+ up-regulated and m_1^- down-regulated genes, respectively. DEGs are selected by t-test. Data replicates: 20.

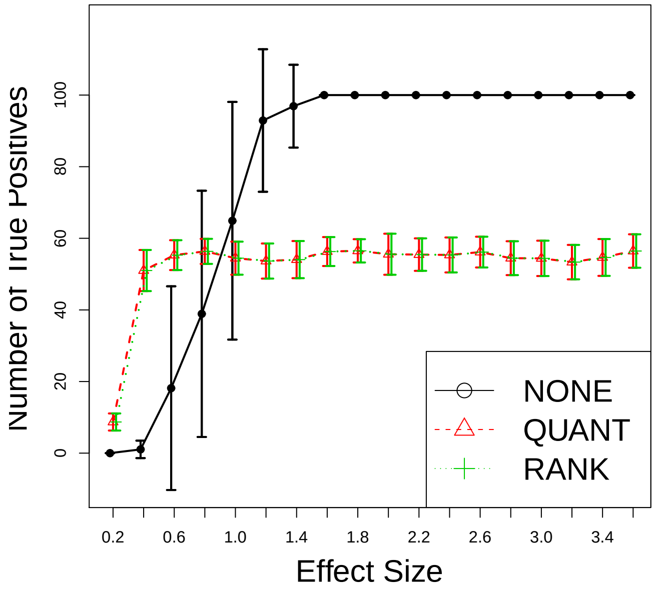

(a) $m_1^+ = 90, m_1^- = 10, n = 10$

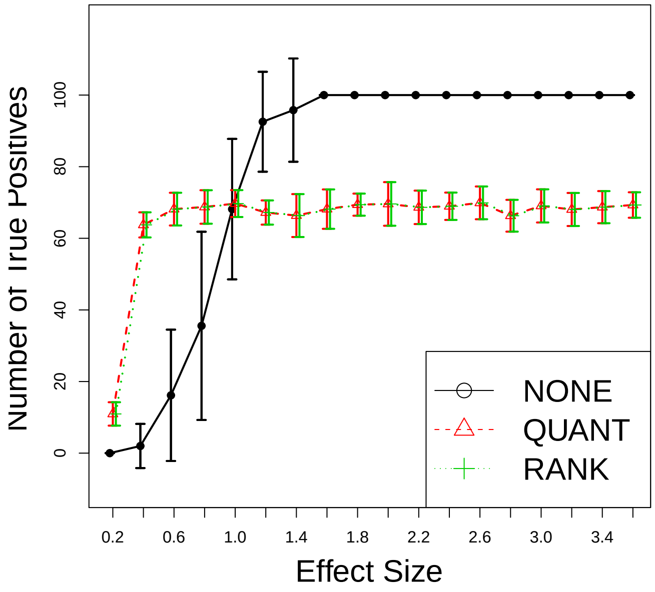

(b) $m_1^+ = 60, m_1^- = 40, n = 10$

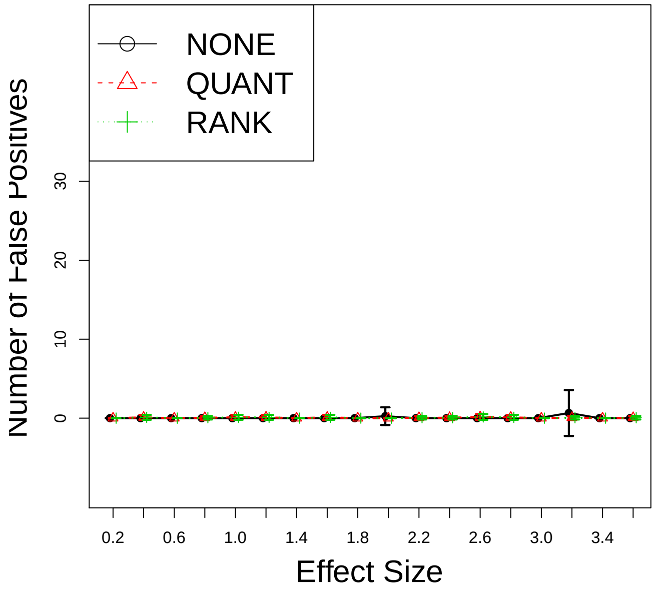

(c) $m_1^+ = 90, m_1^- = 10, n = 10$

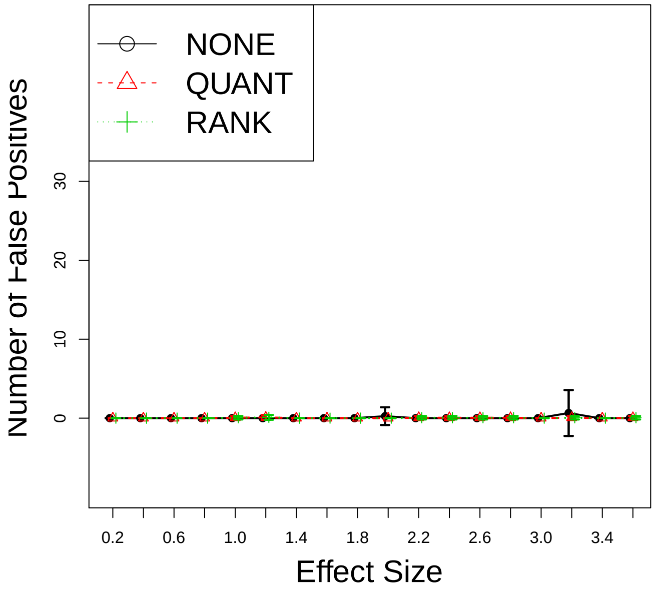

(d) $m_1^+ = 60, m_1^- = 40, n = 10$

Figure 6: Average number of true and false positives as functions of effect size for **SIMU**. The error bar represents one standard deviation above and below average. Total number of truly differentially expressed genes is 100 with m_1^+ up-regulated and m_1^- down-regulated genes, respectively. DEGs are selected by Wilcoxon rank-sum test. Data replicates: 20.

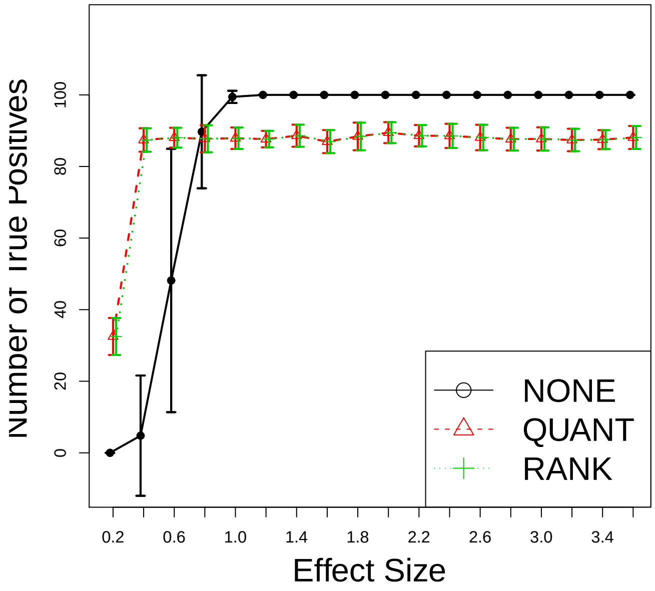

(a) $m_1^+ = 90, m_1^- = 10, n = 15$

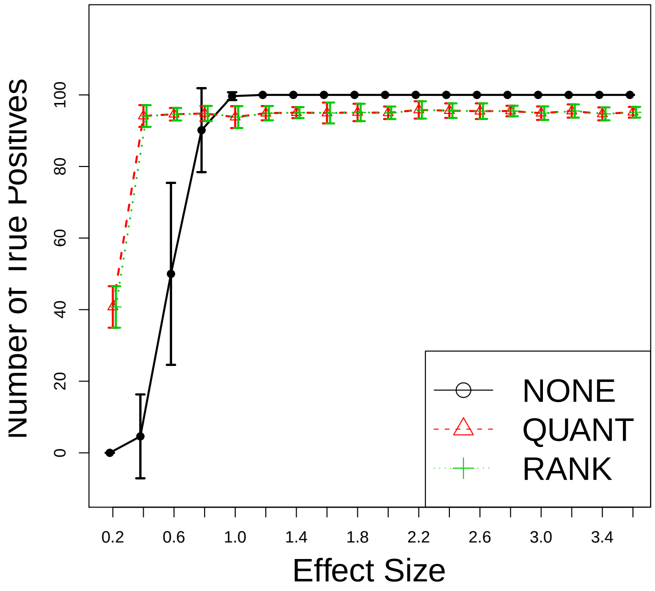

(b) $m_1^+ = 60, m_1^- = 40, n = 15$

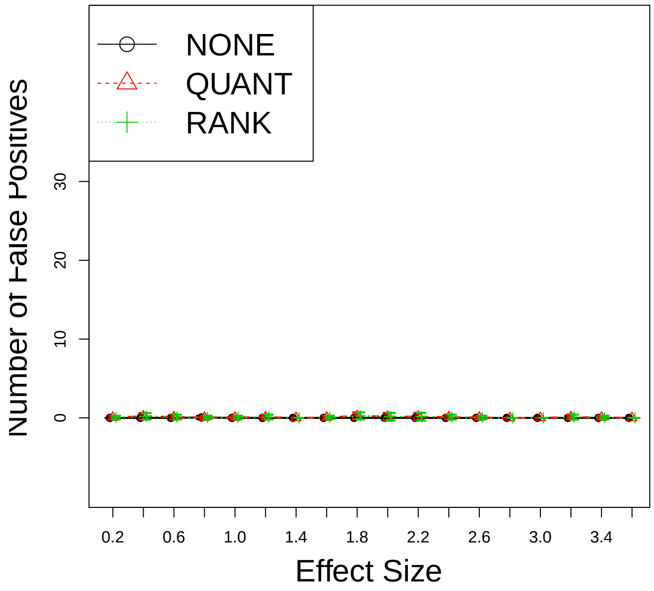

(c) $m_1^+ = 90, m_1^- = 10, n = 15$

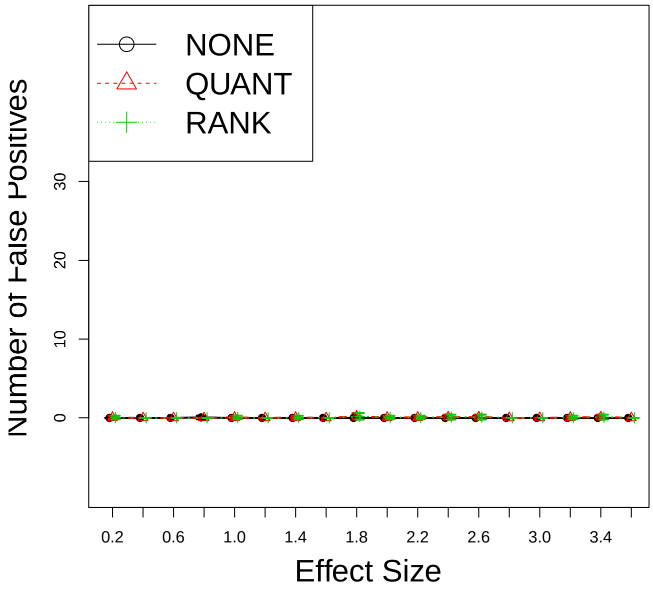

(d) $m_1^+ = 60, m_1^- = 40, n = 15$

Figure 7: Average number of true and false positives as functions of effect size for **SIMU**. The error bar represents one standard deviation above and below average. Total number of truly differentially expressed genes is 100 with m_1^+ up-regulated and m_1^- down-regulated genes, respectively. DEGs are selected by Wilcoxon rank-sum test. Data replicates: 20.

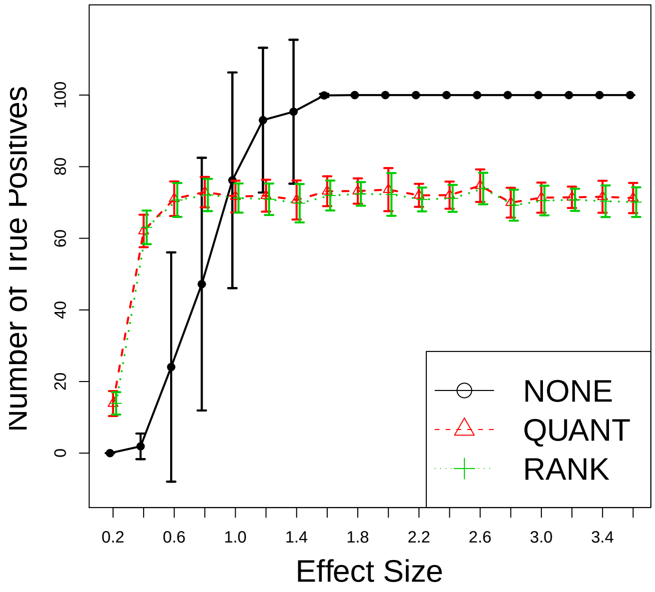

(a) $m_1^+ = 90, m_1^- = 10, n = 10$

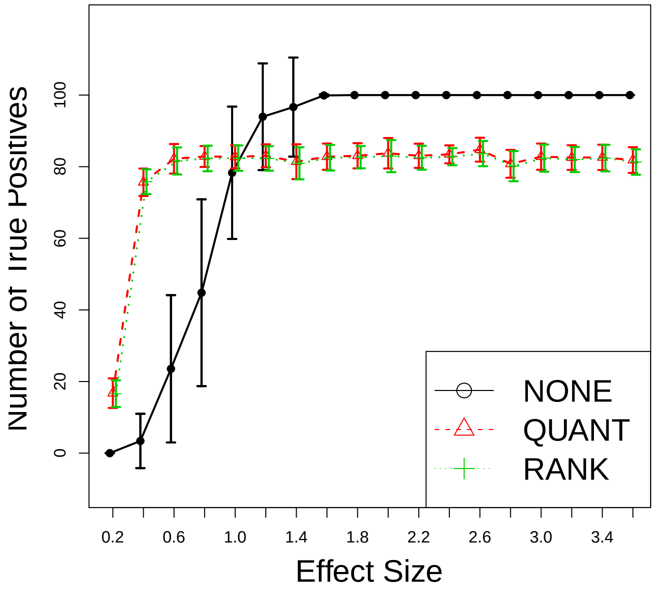

(b) $m_1^+ = 60, m_1^- = 40, n = 10$

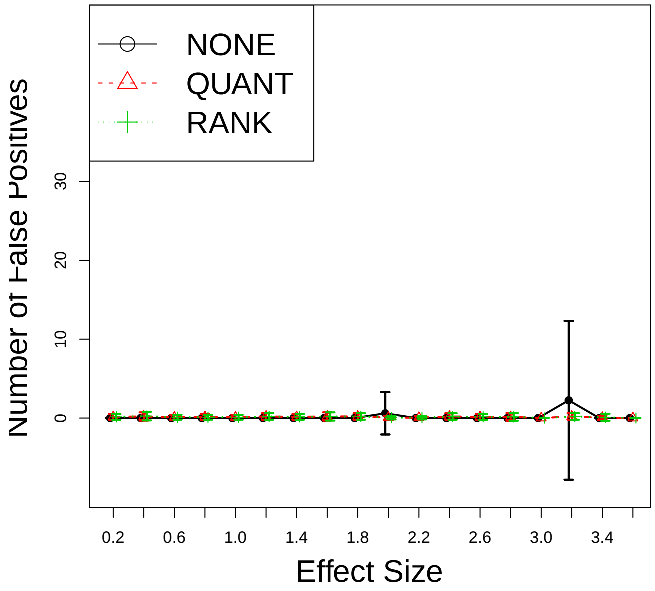

(c) $m_1^+ = 90, m_1^- = 10, n = 10$

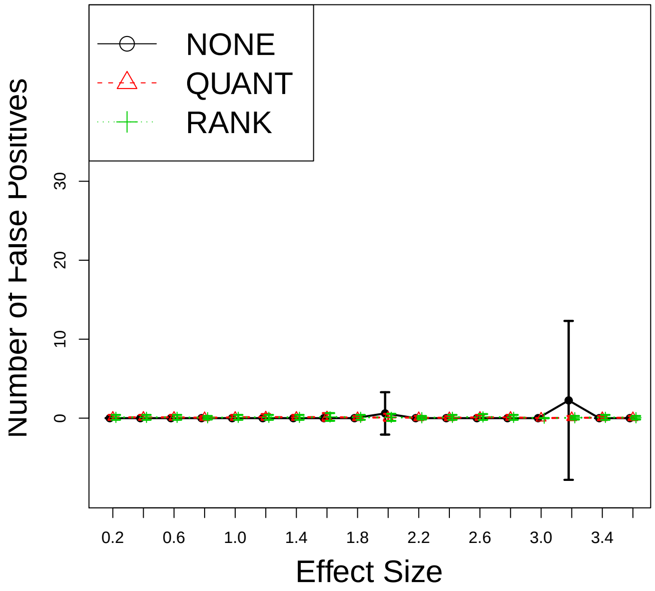

(d) $m_1^+ = 60, m_1^- = 40, n = 10$

Figure 8: Average number of true and false positives as functions of effect size for **SIMU**. The error bar represents one standard deviation above and below average. Total number of truly differentially expressed genes is 100 with m_1^+ up-regulated and m_1^- down-regulated genes, respectively. DEGs are selected by permutation N-test. Data replicates: 20. Number of permutations: 10,000.

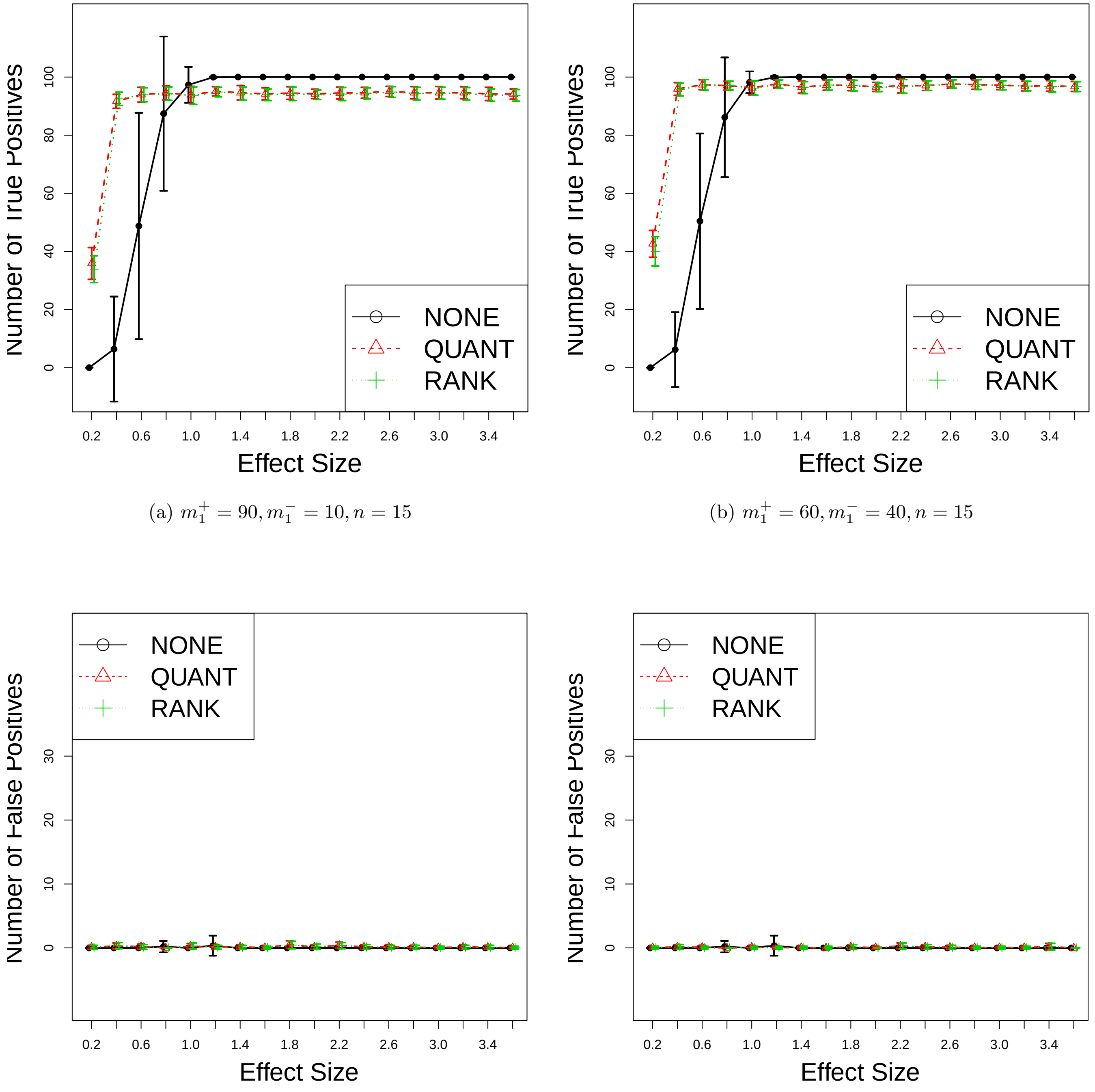

(a) $m_1^+ = 90, m_1^- = 10, n = 15$

(b) $m_1^+ = 60, m_1^- = 40, n = 15$

(c) $m_1^+ = 90, m_1^- = 10, n = 15$

(d) $m_1^+ = 60, m_1^- = 40, n = 15$

Figure 9: Average number of true and false positives as functions of effect size for **SIMU**. The error bar represents one standard deviation above and below average. Total number of truly differentially expressed genes is 100 with m_1^+ up-regulated and m_1^- down-regulated genes, respectively. DEGs are selected by permutation N-test. Data replicates: 20. Number of permutations: 10,000.

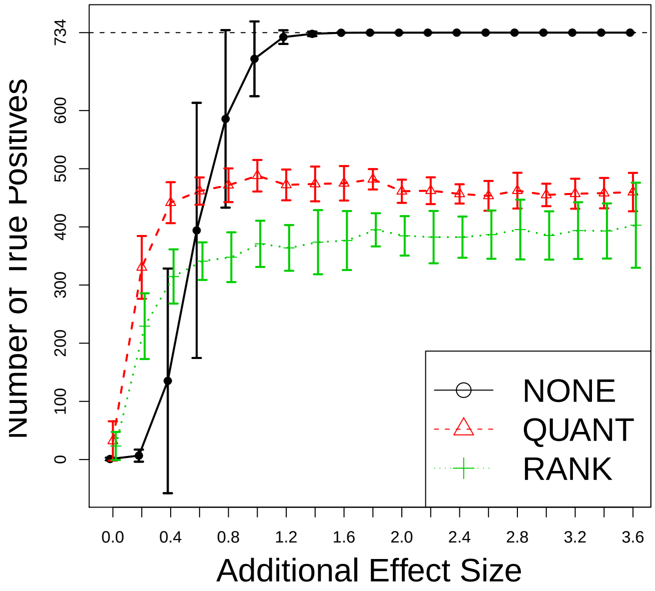

(a) $m_1^+ = 677, m_1^- = 57, n = 15$

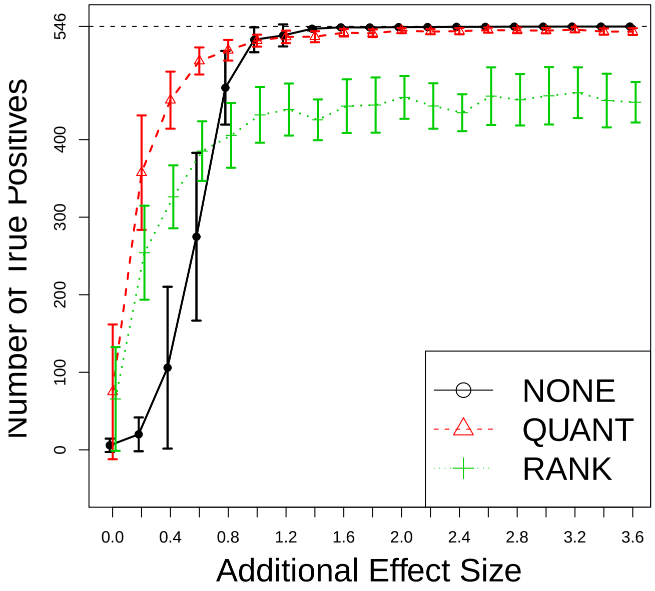

(b) $m_1^+ = 259, m_1^- = 287, n = 15$

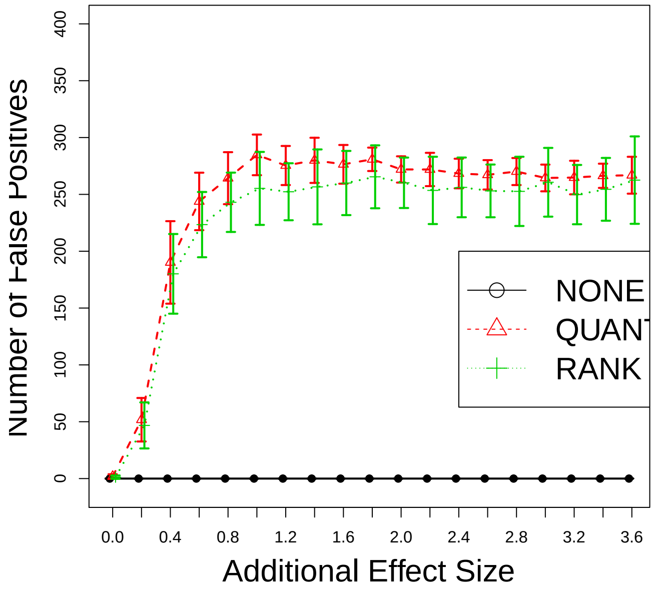

(c) $m_1^+ = 677, m_1^- = 57, n = 15$

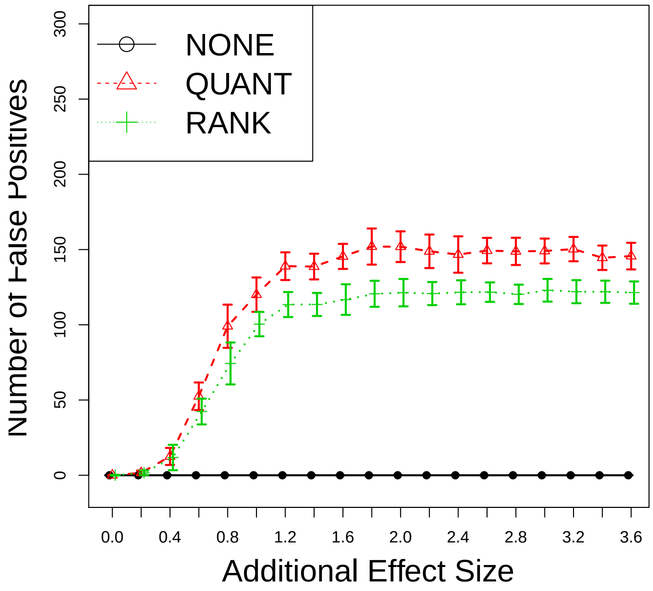

(d) $m_1^+ = 259, m_1^- = 287, n = 15$

Figure 10: Average number of true and false positives as functions of effect size for **SIMU-BIO**. The error bar represents one standard deviation above and below average. Total number of truly differentially expressed genes is $m_1^+ + m_1^1$ with m_1^+ up-regulated and m_1^- down-regulated genes, respectively. DEGs are selected by t-test. Data replicates: 20.

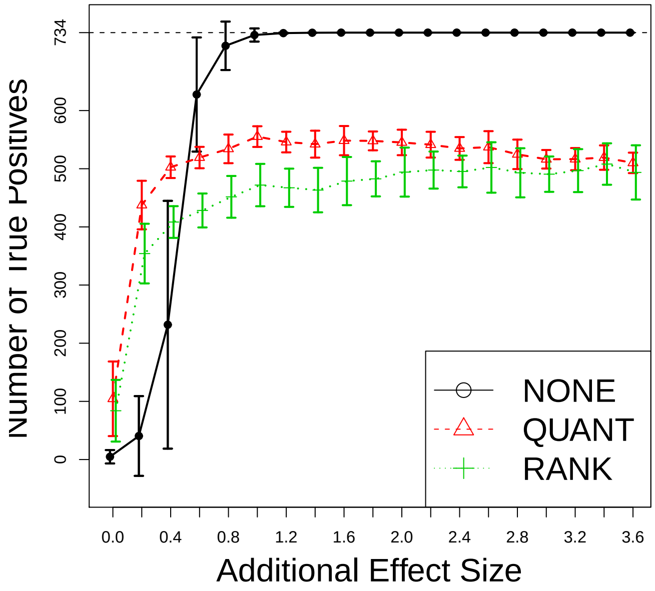

(a) $m_1^+ = 677, m_1^- = 57, n = 20$

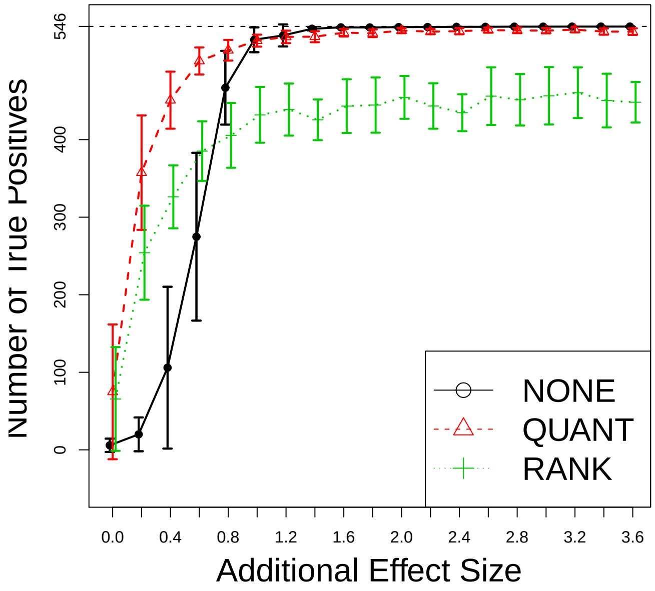

(b) $m_1^+ = 259, m_1^- = 287, n = 20$

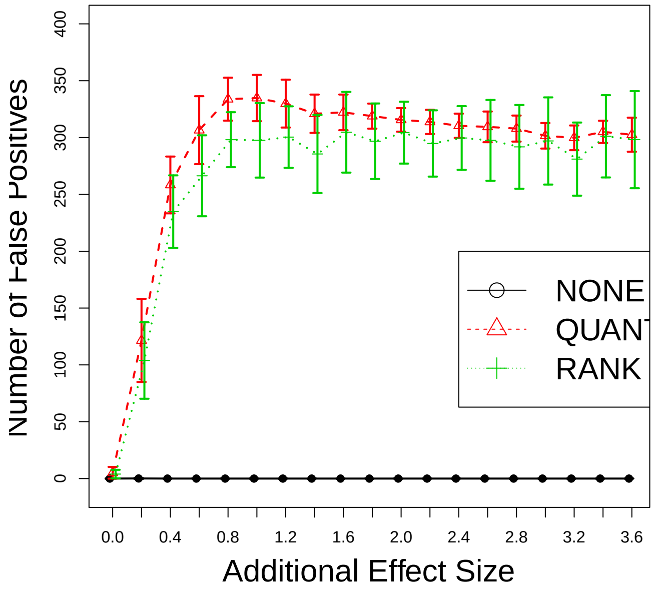

(c) $m_1^+ = 677, m_1^- = 57, n = 20$

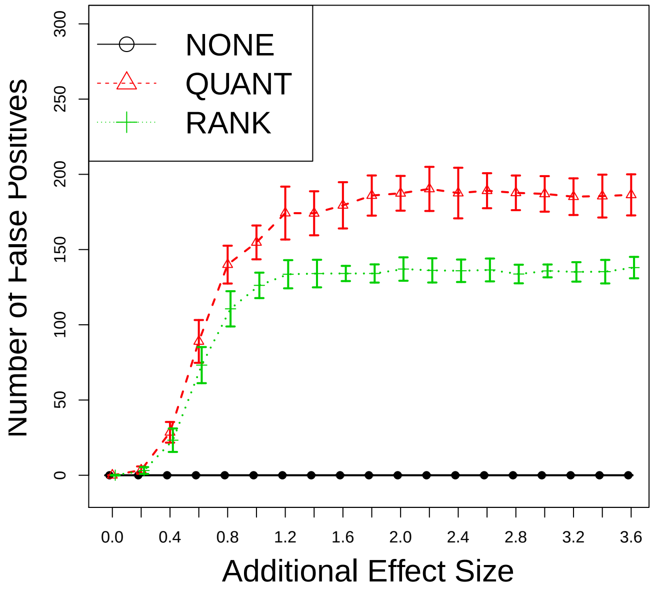

(d) $m_1^+ = 259, m_1^- = 287, n = 20$

Figure 11: Average number of true and false positives as functions of effect size for **SIMU-BIO**. The error bar represents one standard deviation above and below average. Total number of truly differentially expressed genes is $m_1^+ + m_1^1$ with m_1^+ up-regulated and m_1^- down-regulated genes, respectively. DEGs are selected by t-test. Data replicates: 20.

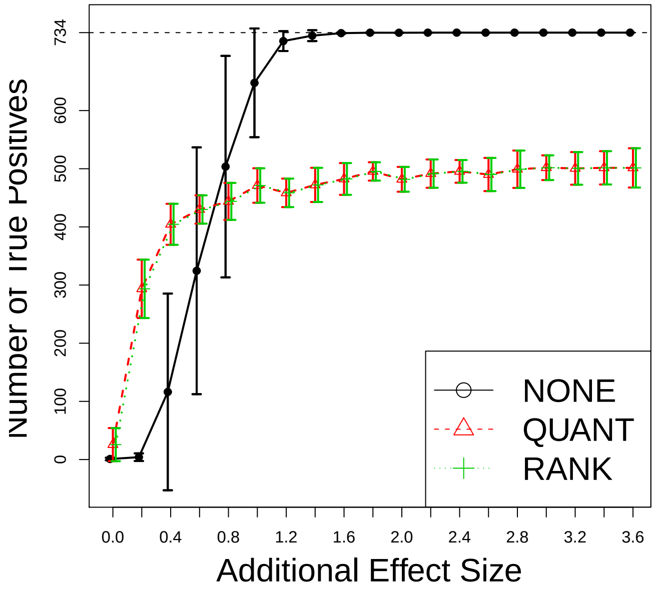

(a) $m_1^+ = 677, m_1^- = 57, n = 15$

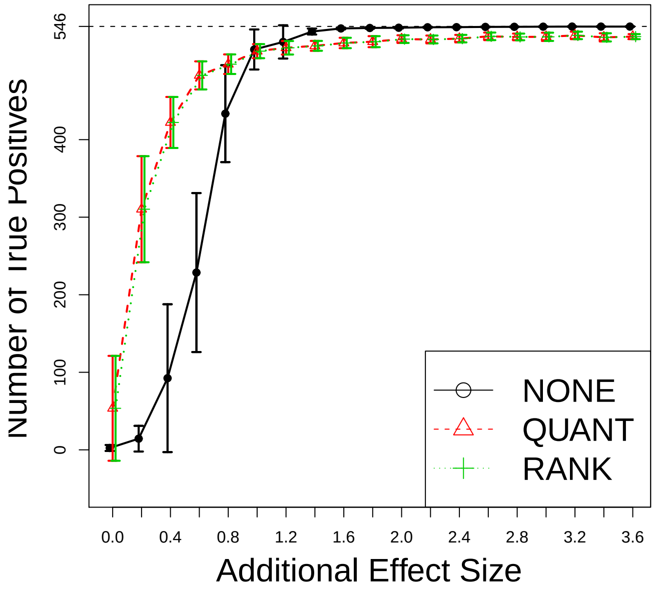

(b) $m_1^+ = 259, m_1^- = 287, n = 15$

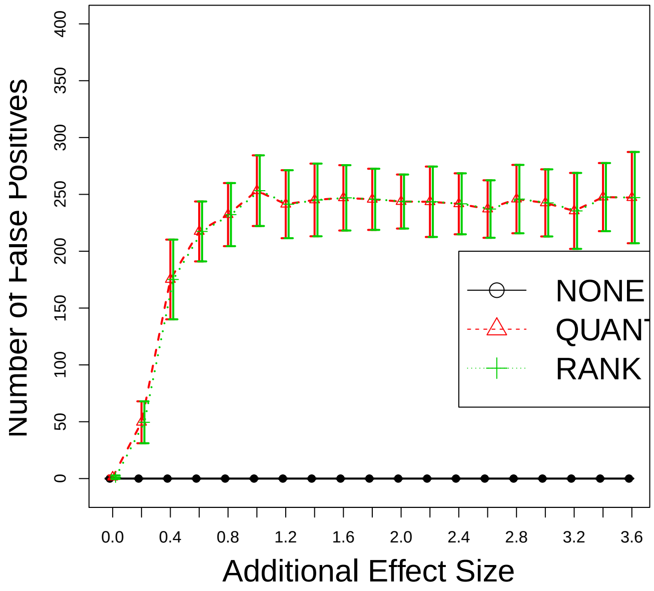

(c) $m_1^+ = 677, m_1^- = 57, n = 15$

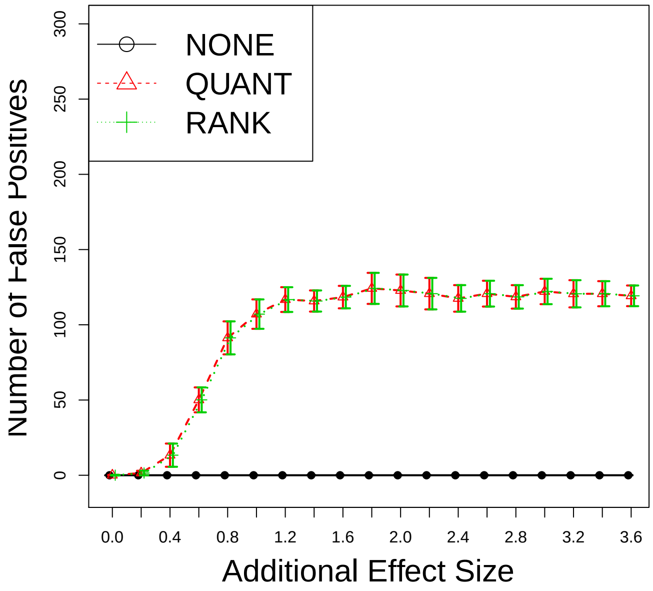

(d) $m_1^+ = 259, m_1^- = 287, n = 15$

Figure 12: Average number of true and false positives as functions of effect size for **SIMU-BIO**. The error bar represents one standard deviation above and below average. Total number of truly differentially expressed genes is $m_1^+ + m_1^1$ with m_1^+ up-regulated and m_1^- down-regulated genes, respectively. DEGs are selected by Wilcoxon rank-sum test. Data replicates: 20.

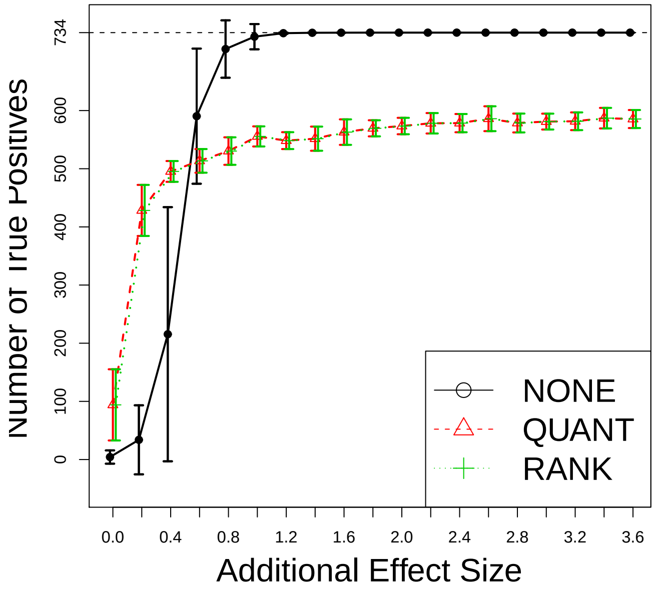

(a) $m_1^+ = 677, m_1^- = 57, n = 20$

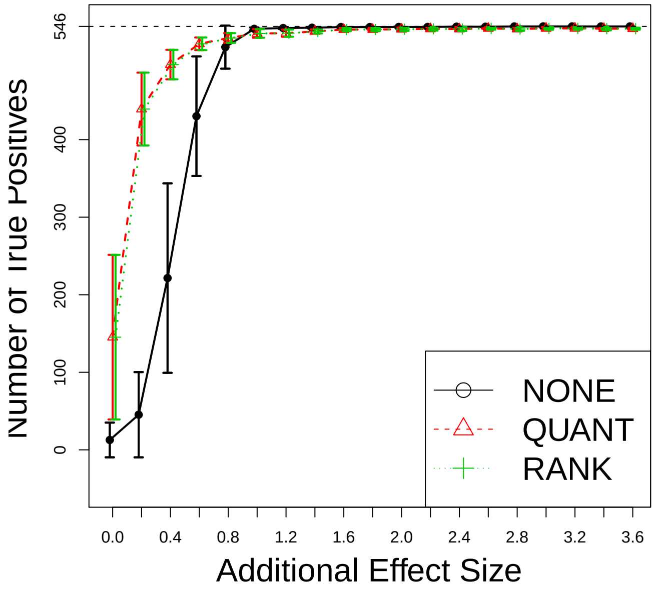

(b) $m_1^+ = 259, m_1^- = 287, n = 20$

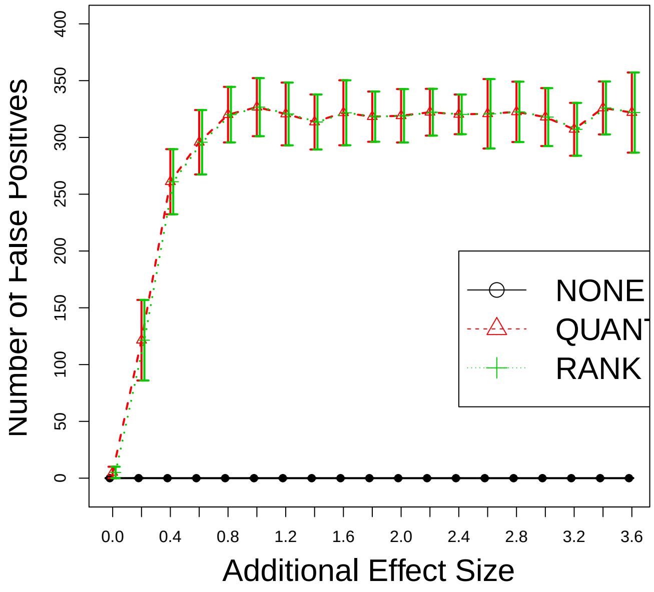

(c) $m_1^+ = 677, m_1^- = 57, n = 20$

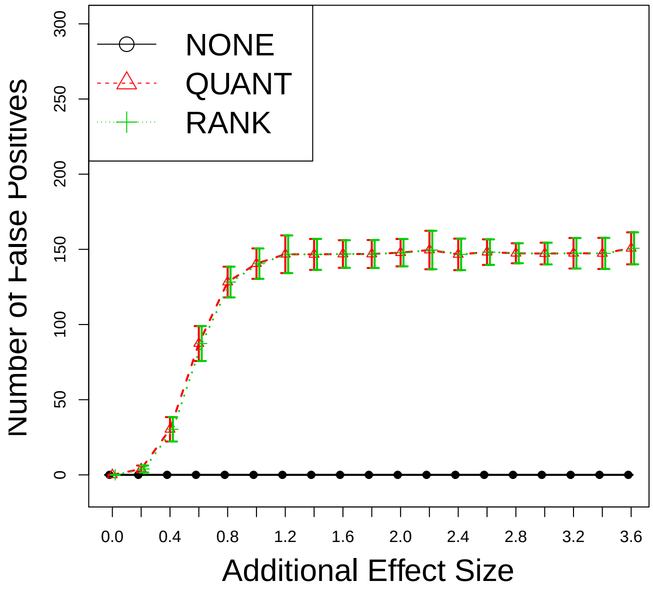

(d) $m_1^+ = 259, m_1^- = 287, n = 20$

Figure 13: Average number of true and false positives as functions of effect size for **SIMU-BIO**. The error bar represents one standard deviation above and below average. Total number of truly differentially expressed genes is $m_1^+ + m_1^1$ with m_1^+ up-regulated and m_1^- down-regulated genes, respectively. DEGs are selected by Wilcoxon rank-sum test. Data replicates: 20.

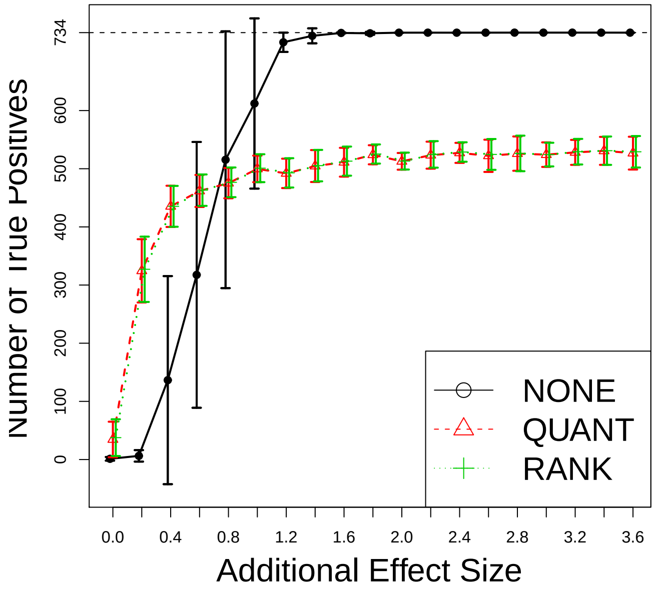

(a) $m_1^+ = 677, m_1^- = 57, n = 15$

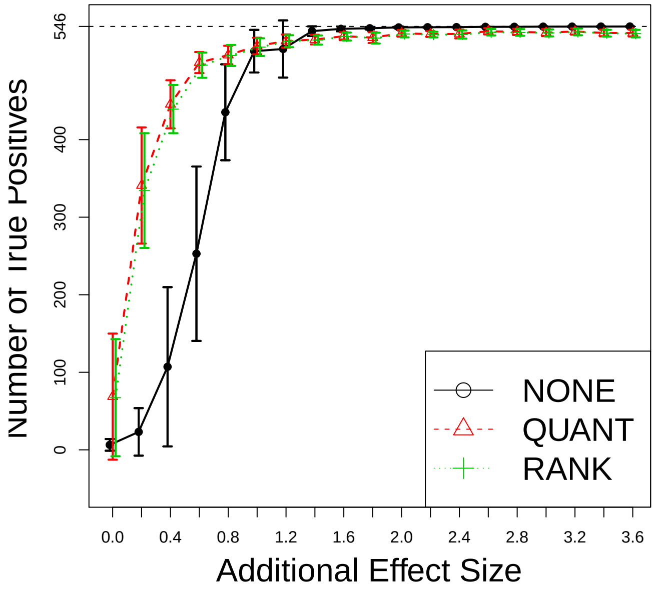

(b) $m_1^+ = 259, m_1^- = 287, n = 15$

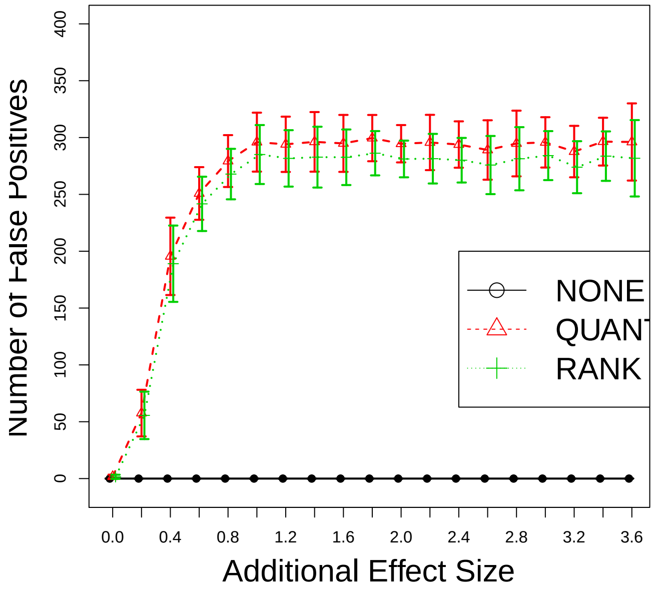

(c) $m_1^+ = 677, m_1^- = 57, n = 15$

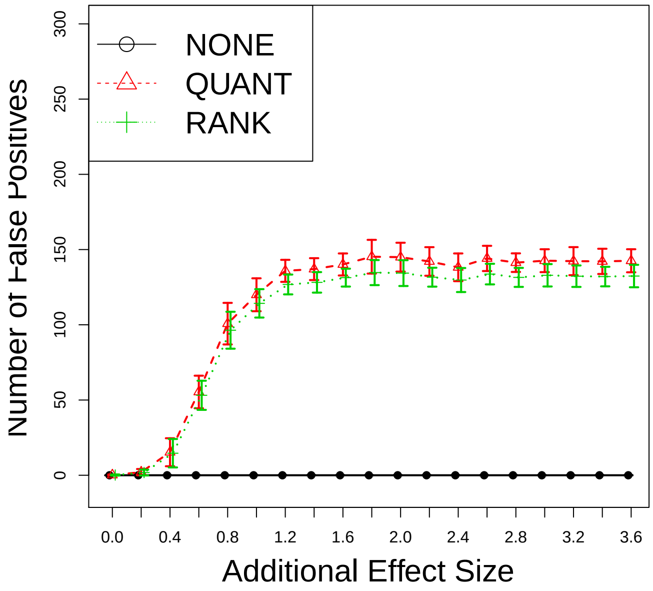

(d) $m_1^+ = 259, m_1^- = 287, n = 15$

Figure 14: Average number of true and false positives as functions of effect size for **SIMU-BIO**. The error bar represents one standard deviation above and below average. Total number of truly differentially expressed genes is $m_1^+ + m_1^1$ with m_1^+ up-regulated and m_1^- down-regulated genes, respectively. DEGs are selected by permutation N-test. Data replicates: 20. Number of permutations: 100,000.

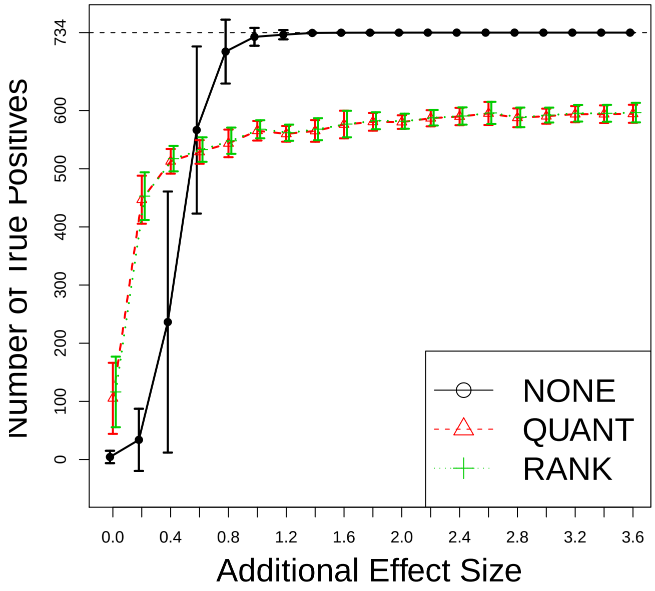

(a) $m_1^+ = 677, m_1^- = 57, n = 20$

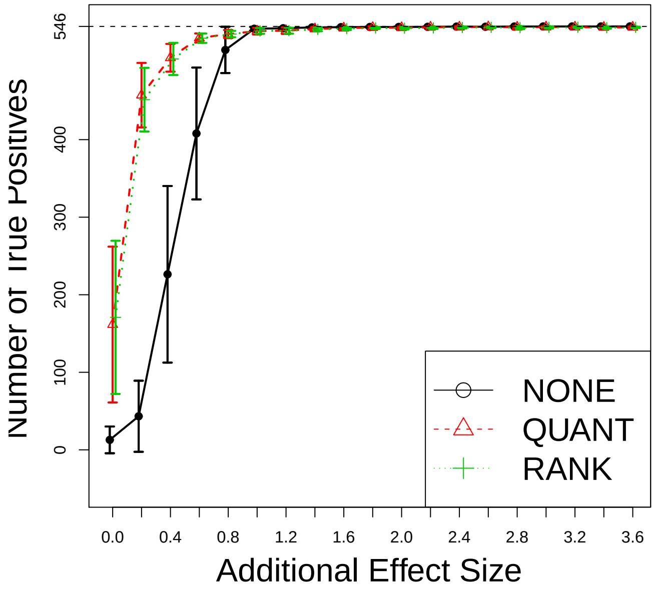

(b) $m_1^+ = 259, m_1^- = 287, n = 20$

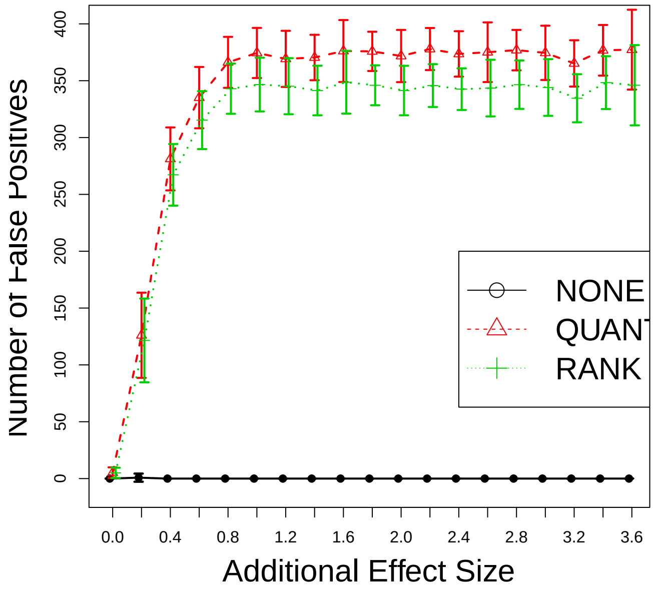

(c) $m_1^+ = 677, m_1^- = 57, n = 20$

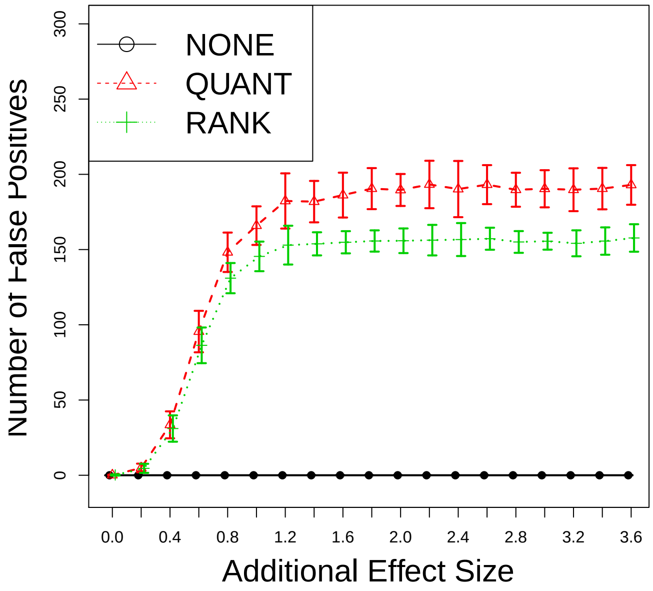

(d) $m_1^+ = 259, m_1^- = 287, n = 20$

Figure 15: Average number of true and false positives as functions of effect size for **SIMU-BIO**. The error bar represents one standard deviation above and below average. Total number of truly differentially expressed genes is $m_1^+ + m_1^1$ with m_1^+ up-regulated and m_1^- down-regulated genes, respectively. DEGs are selected by permutation N-test. Data replicates: 20. Number of permutations: 100,000.

References

[1] Norman L. Johnson, Samuel Kotz, and N. Balakrishnan, *Continuous univariate distributions, volumn 2, second edition*, JOHN WILEY & SONS, INC., 1995.

[2] A. D. MacDonald, *Properties of the confluent hypergeometric function*, Technical report, Research Laboratory of Electronics, Massachusetts Institute of Technology (1948).
